# Amyloid fibril proteomics of AD brains reveals modifiers of aggregation and toxicity

**DOI:** 10.1186/s13024-023-00654-z

**Published:** 2023-09-14

**Authors:** Arun Upadhyay, Deepak Chhangani, Nalini R. Rao, Julia Kofler, Robert Vassar, Diego E. Rincon-Limas, Jeffrey N. Savas

**Affiliations:** 1 Ken and Ruth Davee Department of Neurology, Northwestern University Feinberg School of Medicine, Chicago, IL 60611 USA; 2 Department of Neurology, McKnight Brain Institute, and Norman Fixel Institute for Neurological Diseases, University of Florida, Gainesville, FL 32611 USA; 3 Department of Pathology, Division of Neuropathology, University of Pittsburgh, Pittsburgh, PA 15213 USA; 4 Mesulam Center for Cognitive Neurology and Alzheimer’s Disease, Northwestern University Feinberg School of Medicine, Chicago, IL 60611 USA; 5 Department of Neuroscience, Center for Translational Research in Neurodegenerative Disease, University of Florida, Gainesville, FL 32611 USA; 6 Genetics Institute, University of Florida, Gainesville, FL 32611 USA

**Keywords:** Alzheimer’s disease, Amyloid, *Drosophila*, Fibril purification, Proteomics, Amyloidome

## Abstract

**Background:**

The accumulation of amyloid beta (Aβ) peptides in fibrils is prerequisite for Alzheimer’s disease (AD). Our understanding of the proteins that promote Aβ fibril formation and mediate neurotoxicity has been limited due to technical challenges in isolating pure amyloid fibrils from brain extracts.

**Methods:**

To investigate how amyloid fibrils form and cause neurotoxicity in AD brain, we developed a robust biochemical strategy. We benchmarked the success of our purifications using electron microscopy, amyloid dyes, and a large panel of Aβ immunoassays. Tandem mass-spectrometry based proteomic analysis workflows provided quantitative measures of the amyloid fibril proteome. These methods allowed us to compare amyloid fibril composition from human AD brains, three amyloid mouse models, transgenic Aβ42 flies, and Aβ42 seeded cultured neurons.

**Results:**

Amyloid fibrils are primarily composed by Aβ42 and unexpectedly harbor Aβ38 but generally lack Aβ40 peptides. Multidimensional quantitative proteomics allowed us to redefine the fibril proteome by identifying 20 new amyloid-associated proteins. Notably, we confirmed 57 previously reported plaque-associated proteins. We validated a panel of these proteins as bona fide amyloid-interacting proteins using antibodies and orthogonal proteomic analysis. One metal-binding chaperone metallothionein-3 is tightly associated with amyloid fibrils and modulates fibril formation in vitro*.* Lastly, we used a transgenic Aβ42 fly model to test if knock down or over-expression of fibril-interacting gene homologues modifies neurotoxicity. Here, we could functionally validate 20 genes as modifiers of Aβ42 toxicity in vivo.

**Conclusions:**

These discoveries and subsequent confirmation indicate that fibril-associated proteins play a key role in amyloid formation and AD pathology.

**Supplementary Information:**

The online version contains supplementary material available at 10.1186/s13024-023-00654-z.

## Background

Amyloid beta (Aβ) peptides accumulate, rapidly oligomerize, and can form large degradation-resistant insoluble fibers in Alzheimer’s disease (AD) brains. Aβ peptides are generated by sequential proteolytic cleavage of the amyloid precursor protein (APP) with Aβ38, 40, and 42 being most common. Late-stage AD brains are loaded with Aβ42 peptides that accumulate in a wide range of heterogeneous structures, while Aβ40 peptides are less prone to accumulate [1, 2]. The relevance of Aβ38 peptides is less clear and they may play context-dependent roles in influencing Aβ42 and Aβ40 oligomerization as well as Aβ42 toxicity [3–5]. Aβ oligomeric assemblies frequently coalesce into protofibrils, and can subsequently mature into fibrils that form amyloid plaques [6]. The importance of soluble Aβ oligomers in the etiology of AD is well established, while the precise role of Aβ fibrils in AD pathogenesis remains unclear [7–9]. Nonetheless, several studies have confirmed that insoluble fibrils can exert toxicity, contribute to synaptic dysfunction, microglial activation, and neurodegeneration in AD brains [10–12]. Determining the mechanisms responsible for amyloid fibril formation may provide new and relevant insight into the development of therapeutic strategies for reducing the amyloid load.

The relevance of amyloid fibrils in AD is highlighted by the recent therapeutic success of Lecanemab, which preferentially binds to large, soluble Aβ protofibrils [13]. However, the complex biochemical properties of protofibrils (e.g., size distribution, solubility, and degree of hydrophobicity), have presented a barrier to our understanding of these toxic proteinaceous assemblies [14]. Fibrils represent end point structural assemblies in the long process through which Aβ monomers can gradually accumulate into large aggregates and form mature plaques [15, 16]. It’s possible that inhibiting fibril formation or maturation could reduce the amyloid burden, restore proteostasis, and even prevent neuronal death. However, thus far several technical limitations have limited our ability to study AD brain-derived fibrillar assemblies, determine their composition, and physiological impact. The most significant obstacle has been our inability to obtain highly purified amyloid fibrils from AD brain tissue extracts [17]. To circumvent this requirement, amyloid fibril structure has primarily been studied using synthetic Aβ peptides seeded with AD brain isolates [18, 19]. These seeding experiments produce a variety of amyloid structures but precisely how they relate to fibrils formed in the brain is unclear. Recently, several groups have succeeded in isolating highly pure amyloid fibrils from mouse and human brains and solved their structures [20, 21]. However, an exhaustive proteomic composition of these fibrils beyond Aβ peptides has yet to be reported.

The formation of amyloid fibrils in the brain is a complex process that requires long time frames and culminates in the deposition of plaques predominantly near synapses in the extracellular space [15]. A variety of proteins have been found trapped in or aggregated near plaques, but direct and indirect amyloid fibril-binding proteins are largely unknown. Previous mass spectrometry (MS)-based proteomic analyses of the Aβ interactome or the amyloid plaque proteome have reported hundreds or even thousands of proteins. Most of these studies used traditional biochemical approaches, laser microdissection, or affinity purification and captured a heterogeneous pool of amyloid-associated or coprecipitated proteins from brain, blood, or cerebrospinal fluid [22–24]. Despite these efforts, we still lack a clear understanding of the proteins involved in amyloid fibril formation and stabilization. This is mainly due to the large number of proteins and the inconsistent pool of identified proteins.

To identify proteins influencing amyloid fibril formation or modulating toxicity, we developed an amyloid fibril core purification strategy and used leading MS-based analyses to determine their content. Detailed inspection of the Aβ peptide isoforms in the amyloid fibrils revealed the purified fibrils predominantly contained Aβ42, and Aβ38, while Aβ40 was the least abundant variant. In vitro peptide-based studies showed Aβ38 can accelerate Aβ42 fibril formation. Inside the brain, there could be other proteins present in low concentrations in the proximity of Aβ peptides influencing their aggregation and cross-reactivities. Our comprehensive proteomic analyses revealed a consistent panel of proteins associated with amyloid fibrils purified from multiple biological sources, including postmortem AD patient brains, three mouse models of amyloid pathology, Aβ42 overexpressing flies, and cultured neurons seeded with Aβ42 peptides. A panel of selected proteins were verified with antibodies. Among the top candidates was metallothionein-3 (MT3), which influences Aβ42 aggregation in vitro*.* Finally, we confirmed that several of these proteins also regulate Aβ42-induced toxicity in a *Drosophila* model. Taken all together, our study provides a pioneering description of AD amyloid fibrils and elucidates the functional influence of a panel of Aβ-interacting proteins on fibril formation and in vivo toxicity.

## Methods

### Animals

A total of four mouse models were used: transgenic 5xFAD, and three App knock in (*App* KI) mouse models: *App*^*NL/NL*^*, App*^*NL−F/NL−F*^*, and App*^*NL−G−F/NL−G−F*^ [25, 26]. A detailed description of the mutations and pathological features of these mouse models is provided in Additional file 2. Animal care and experimental protocols in this study were designed and performed as per National Institutes of Health Guidelines. Northwestern University’s Institutional Animal Care and Use Committee (IACUC) approved the protocol (protocol IS0009991). For stable ^15^N isotope labeling, previously described method was followed for labeling WT animals [27]. Briefly, animals were kept on ^15^N enriched Spirulina-based diet (obtained from Cambridge Isotopes Laboratories) for three months starting at P28. For euthanasia, mice were anesthetized with 3% isoflurane followed by cervical dislocation and acute decapitation. Required brain regions for each experiment were harvested, flash-frozen in a dry ice/ethanol bath, and stored at − 80 °C.

### Human samples

Frozen post-mortem frontal cortex tissue was obtained from the University of Pittsburgh neurodegenerative brain bank. Brain tissues were donated with consent from family members of the AD patients and approval of the University of Pittsburgh Committee for Oversight of Research and Clinical Training Involving Decedents. All institutional guidelines were followed during the collection of tissues. Staging of AD pathology was performed using NIA-AA criteria [28]. Additional details on AD patients their diagnosis, and neuropathological conditions are provided in Supplementary Table S1.

### Amyloid fibril purification from brain tissues

Biochemical purification of amyloid fibrils from mouse and human brain tissues was performed using novel technological modifications in methods described previously [29, 30]. Freshly harvested or snap-frozen brain tissues (0.25 -1 g) were homogenized in 1 ml buffer A (0.25 M sucrose, 3 mM EDTA, 0.1% sodium azide, and protease inhibitor cocktail in 10 mM Tris–HCl pH 7) and solubilized overnight with end-to-end rotation. For *Drosophila,* heads from flies expressing either LacZ (control) or Aβ42 using 201Y-Gal4 driver combined with nls-mcherry were snap-frozen. Before purification, fly heads were pooled into groups of sixty heads each and homogenized in equivalent volume of buffer A. The next day, by adding dry sucrose powder, the sucrose concentration was raised to 1.2 M. The solubilized tissue homogenate was then centrifuged for 45 min at 250,000 × g, 4 °C. After discarding the top whitish layer and intermediate aqueous layers, the pellet was dispersed in the same volume Buffer A with a higher 1.9 M sucrose concentration. Next centrifugation was done for 30 min, 125,000 × g, at 4 °C. The pellet is washed twice in 1 ml wash buffer (50 mM Tris–HCl) by rotating at 8,000 × g, 4 °C for 15 min. Digestion buffer containing collagenase and DNase I is added to solubilize and digest the pellet for three to four hours at 37 °C and washed again in the same Tris–HCl buffer. Following this, the pellet is immediately dissolved in 1 ml buffer A with 1.3 M sucrose and 1% SDS. Next, solubilized pellets were centrifuged for an hour at 200,000 × g, 4 °C. Pellet is saved on ice and the supernatant is centrifuged again with reduced sucrose concentration (up to 1 M), at 250,000 × g for 45 min. Both pellets were combined and dissolved in 200 µl Tris buffer. The aqueous solution containing highly enriched amyloid material is subjected to water bath ultrasonication in Bioruptor Pico Plus (15 cycles, medium frequency) and washed five times in Tris buffer containing 1% SDS at 16,000 × g, 20 min, 4 °C. The final pellet is saved and dissolved in 100 µl MilliQ water or buffers per experimental requirements.

### Amyloid fibril purification from seeded primary neurons

Primary hippocampal neurons were cultured from embryonic E18 rats (Envigo). Neurons were dissociated in Papain and plated on poly-D-lysine (Sigma-Aldrich #P0899) and laminin (Gibco™ 23017015)-coated plates. Neurons were kept in Neurobasal media (Gibco™ 21103049) supplemented with SM1 (STEMCELL Technologies #05711), glutamax (Gibco™ A1286001), filtered glucose, and β-mercaptoethanol (Thermo Scientific # 0219483425) and maintained for 21 days. At DIV 21, neurons were seeded with 10 µM recombinant Aβ42 fibrils (rPeptide A-1163–2). Preformed assemblies were sonicated for 20 min in a water bath sonicator before seeding. Following incubation, cells were collected in the media using cell scrapers, and the above-described strategy was used to purify amyloid fibrils.

### Immunoblots

For WB, protein concentrations in each sample were measured with BCA protein Assay Kit (Thermo Scientific, Cat# 23225). Equal quantities of protein samples were boiled for five minutes in SDS Laemmli buffer. Samples were immediately loaded onto the 4–15% Mini-PROTEAN TGX Stain-free precast gels (BioRad # 4568084) and electrophoresed for high-resolution separation of proteins based on the size. Following the electrophoresis run, the gels were used for Coomassie brilliant blue or silver staining to visualize the complete protein profile in each sample. Alternatively, transfer of total protein content onto a 0.45-micron size nitrocellulose membrane was achieved in a Bio-Rad semi-dry quick transfer apparatus. Before blocking the membranes with 5% milk, ponceau S (Sigma Aldrich #P7170), a reversible protein binding stain, was used to observe the profile of membrane-bound proteins. After 60 min of blocking at RT, membranes were incubated overnight at 4 °C in a required concentration of primary antibodies prepared in Tris-buffered saline with 0.1% Tween®20 (TBST). The next day, following four washes in TBST, five minutes each with shaking, membranes were probed with HRP-conjugated secondary antibodies obtained from the same host. Following four TBST washes, chemiluminescence was recorded under the Bio-Rad ChemiDoc® MP Imaging system. Similarly, we performed membrane-trap dot blot analysis using a previously described method [31]. In brief, an equal amount of protein from each sample were blotted manually on pre-activated membranes and blocked with a 5% milk solution prepared in TBST. Ponceau S staining was used for visualizing the loaded protein amount. Antibody incubation, washing, and chemiluminescence detection were performed similar to WB.

### Immunostaining / immunohistochemistry

Perfusion, sectioning, and immunohistochemistry were performed as previously described [32]. Briefly, mice were transcardially perfused with PBS and drop-fixed in 4% paraformaldehyde for 24 h. Fixed brains were then cryoprotected in 30% sucrose for at least 2 days before being embedded in Tissue-Tek OCT Compound for cryostat sectioning. Sagittal sections were prepared at 25–35 μm thickness and mounted onto gelatin-coated slides (Southern Biotech, Cat# SLD01-CS). For immunostaining, sections were kept at RT for 2 h and then washed with PBS (3 × 5 min) to remove OCT. Sections were then blocked and permeabilized with 0.2% Triton-X 100 and 10% Horse Serum (HS) in PBS for 3 h at RT. After three PBS washes, sections were incubated overnight at 4c with primary antibodies diluted in 1% HS and 0.1% Triton-X 100. The next day, sections were washed with PBS (3 × 5 min) and then incubated with secondary antibodies in PBS. After secondary antibody incubation, sections were washed with PBS (3 × 5 min) and coverslips were mounted with Fluoromount-G. Images were taken using a Nikon AXR confocal microscope at 10 × and 63x.

For immunostaining of purified material, the fibrils were washed three times in 1% PBS before being blocked in 2% horse serum. After two PBS washes, fibrils were incubated overnight at 4 °C with primary antibodies dissolved in PBS with 0.2% serum. The next day, fibrils were washed three times and incubated with fluorescent secondary antibodies. 10 µL of each sample were put on glass slides and observed under a Leica confocal microscope with a 63 × oil objective.

### Congo red staining

Staining of fresh amyloid preparations was performed by incubating the samples with filtered 0.1% Congo red (Sigma Aldrich #C6277) solution, prepared in 50% ethyl alcohol for 20 min at RT. The stained samples were observed by microscopy using bright field illumination and cross-polarized light separately at 40X magnification.

### Amyloid kinetics experiment

For the ThT-based kinetic analyses, 10 mM ThT stock solution was prepared in 1% PBS and filtered through a 0.2-micron syringe filter. Before starting the kinetic experiments, the recombinant Aβ peptides (Aβ38, rPeptide A-1078–2; Aβ40: rPeptide A-1153–2; Aβ42: rPeptide A-1163–2 and Aβ42_scrambled_, rPeptide A-1004–02) were solubilized and denatured into monomers with HFIP and 6 M GuHCl. Next, they were diluted in aggregation buffer (PBS pH7.4, Growcells, cat.#: MRGF6396), ultrasonicated for 20 min at 10 °C and centrifuged at 10,000 × g for 5 min to remove any remaining aggregates (refer Fig. S3a). The aggregation reactions (100 µL / well) were set up with 3 µM Aβ peptides and 20 µM ThT in the aggregation buffer using 96-well plates. Additional blank wells were set up without ThT or Aβ peptides. The program in the plate reader was created to read (excitation wavelength: 440 nm, emission wavelength: 482 nm) the emission every four minutes for next three hours.

For two-peptide experiments, the additional Aβ peptides were added at 100 nM with 3 µM Aβ38, Aβ40 and Aβ42 peptide solutions. We used Aβ42_scrambled_ peptides as negative controls for these experiments. ThT fluorescence was recorded every 4 min. For MT3 experiment, 100 nM recombinant human MT3 protein (Boster Bio Cat no. PROTP25713) was incubated with 3 µM Aβ42. ThT flouroscence was recorded every four minutes. For all the experiments, total aggregate concentrations were calculated using the secondary nucleation-dominated model in the AmyloFit online tool (https://amylofit.com). The kinetic (fit) curve values obtained from Amylofit were plotted in Graphpad. Values plotted on graph are independent values obtained from three to ten replicates for each reaction condition.

### ELISA assay

Aβ38 (IBL Amarica #27717), Aβ40 (Thermo Fisher #KHB3781), and Aβ42 (Thermo Fisher #KHB3441) ELISA analyses, were performed in 96-well plates per the manufacturer’s instructions. For the Aβ peptide enzyme-linked immunosorbent assay (ELISA) analysis (Aβ38, 40, and 42) the purified fibrils were solubilized in 5 M GuHCl for 2 h with sonication and vortexing at RT. Samples were then diluted 1:60 for *App*^*NL/NL*^; 1:300 for *App*^*NL−F/NL−F*^, 1:600 for *App*^*NL−G−F/NL−G−F*^, and 1:600 for 5xFAD in the standard diluent buffer. Similarly, the control, AD (A2 and A3) human brain fibril samples were diluted 1:60, 1:500, and 1:1500, respectively. The same amount of GuHCl was also added to the Aβ peptide standards and blank measurements. 50 μL of blank solution, standards, and samples were loaded into antibody-coated wells and incubated with detection antibody for 3 h at RT. After three washes in 1X wash buffer (provided in kits), HRP-conjugated antibody was added for 30 min. After three washes, the samples were incubated with stabilized chromogen for 30 min, and the reaction was stopped with an acid-based stop solution. Finally, OD was measured at 450 nm using a Synergy HTX multimode microplate reader (Biotek) and compared to a standard curve to determine the final concentration.

For aggregated Aβ ELISA (Thermo Fisher #KHB3791), a similar 96-well plate was prepared (but without GuHCl) using 100 μL of blank, standard, and diluted test samples in a pre-coated plate with anti-Aβ aggregate antibody, which primarily captures oligomeric aggregates, but also shows reactivity for fibrils. After two hours, thoroughly washed wells were incubated for an hour with human aggregated Aβ biotin conjugate solution. Immediately after four washes, thirty minutes of incubation in a streptavidin-HRP working solution were done. After carefully decanting the liquid from each well washed four times, stabilized chromogen was added and stopped after thirty minutes. Finally, OD measurements for each well were taken on a microplate reader.

### Negative staining and immunogold labeling electron microscopy

For negative staining, fibrils were dissolved in MilliQ water, and 10 µL aliquot was adsorbed in duplicate on Formvar/Carbon Supported 200 mesh Copper Grids for 1–2 min. Following blotting, and rinsing with water, grids were immediately stained with 10 μL of 2% w/w uranyl acetate for 30 s. Grids were again blotted and dried in air. Dark-field images were taken with an Eagle 4 k HR 200 kV CCD camera mounted on FEI Tecnai Spirit G2 transmission electron microscope (FEI) operated at 80 kV. For immunogold labeling, sample preparation was done in accordance with established protocols. Fibrils were first incubated with a blocking solution containing 0.1% Tween®20, 1% bovine serum albumin, 1% normal goat serum, and 0.005% sodium azide diluted in Tris-buffered saline (TBS) buffer, pH 7.4. Next, the washed fibrils were incubated with primary antibodies and control IgG antibody at 1:500 dilution for four hours at 4 °C and washed thrice with PBS. Fibril-antibody conjugates were dissolved in PBS and 10 μL solution was used for adsorption on the 200 mesh copper grids, followed by incubation with colloidal gold secondary anti-mouse or anti-rabbit secondary antibodies for one hour. Washing with TBS and stabilization with 1% glutaraldehyde for 5 min were performed before counterstaining in uranyl acetate. Images were taken with FEI Tecnai Spirit G2 transmission electron microscope at 80 kV acceleration voltage.

### Proteolysis experiment

For the complete digestion of fibrils, we prepared a cocktail of multiple proteolytic enzymes with distinct specificities and wide footprints. In brief, the protease cocktail consists of α-chymotrypsin (Sigma, Cat#C3142), thermolysin (Sigma, Cat#P1512), endoproteinase Asp-N (New England Biolabs, #P8104S), Glu-C (Sigma, Cat#P2922), Arg-C (Biovendor, Cat#RBG40003005), trypsin (Promega, Cat# V5280), and Lys-C (Promega, Cat# PI90307). In a 50 µL reaction solution, 50 µg fibrils were incubated with continuous mixing with different concentrations (1X, 0.5X, and 0.25X) of protease cocktail. Concentrations of various proteases were standardized and kept in the range of 0.01 to 0.1 µg for each reaction mixture. After 30 min of incubation, reactions were quenched with 2X SDS buffer containing 5.2 mM PMSF and 5.2 mM EDTA, at 95 °C for 5 min. One-fifth by volume of each reaction mix was used for WB analysis, while the rest of the sample was reduced and alkylated before overnight incubation with trypsin for digesting remaining undigested fibril assemblies. The next day, following peptide clean-up, samples were dried and resuspended in peptide resuspension buffer to analyze 3 µg of peptides with label-free MS.

### Genetic validation in Drosophila

To perform functional in vivo validation of our amyloid-associated proteins from MS analysis, we utilized a well-established Drosophila model of extracellular Aβ42 deposition and toxicity [33, 34]. For this, we used a recombinant line that expresses a UAS-Aβ42 transgene in photoreceptor neurons under control of the eye-specific GMR-Gal4 driver. Thus, we crossed these recombinant Aβ42 flies with innocuous LacZ/Luciferase RNAi control transgenes and with RNAi/overexpression lines corresponding to hits from proteomics data. These crosses were cultured at 27 °C throughout development, and then newly eclosed flies were observed under the microscope for phenotypic analysis in the eyes. At least five flies per genotype were randomly selected to acquire multi-focal montage imaging using Leica Z16 Apo zoom system. Transgenes that alleviate Aβ42 toxicity in the eye were categorized as suppressors, while those that make it worse were scored as enhancers. Quantification of eye phenotype was performed manually using severity scores based on eye size, depigmentation, necrosis, and ommatidial disorganization [35].

### MS sample preparation- label free quant

The protein solutions were subjected to traditional chloroform/methanol precipitation, followed by structural denaturation in 50 µL of 8 M urea dissolved in 50 mM ammonium bicarbonate (ABC) buffer. The same volume of 0.2% ProteaseMAX (Promega, Cat# V2072) solution in ABC buffer was added and incubated for an hour with vortex. The disulfide bonds in proteins were reduced with 5 mM Tris(2-carboxyethyl)phosphine (TCEP) for 20 min at RT, followed by alkylation with 10 mM iodoacetamide (IAA). Tubes were incubated in the dark for 15 min and immediately quenched with excess (25 mM) of TCEP prepared in ABC. Subsequently, proteins were digested overnight at 37 °C using MS-grade trypsin (Promega, Cat# V5280). The next morning, digestion reaction was stopped by acidification using 1% formic acid (FA). Desalting using C18 spin columns (Thermo Scientific, Cat# 89,870) was performed per the manufacturer’s instructions. Peptide solutions were dried down in a refrigerated speed vac and stored at − 80 °C.

### Tandem mass tag (TMT)- MS sample preparation

We performed TMT-MS analysis following previously described methods [36]. Briefly, 100 μg of protein for each biological sample was extracted using Methanol chloroform precipitation. The protein pellets were resuspended in 6 M guanidine solution prepared in 100 mM triethylammonium bicarbonate (TEAB) buffer (Thermo Scientific, Cat# 90,114). The protein solutions were reduced with 5 mM dithiothreitol (DTT) and alkylated at free SH groups of cysteine residues with 20 mM IAA. Digestion reaction for proteins was initially set up with 1 μg of MS grade LysC (Promega, Cat# PI90307) for 3 h at RT and then continued overnight with addition of 2 μg of trypsin (Promega, Cat# V5280), at 37 °C. The next morning, the digest was acidified and desalted using C18 HyperSep columns (Thermo Fisher Scientific, Cat# 60,108–302). The eluted peptide solution was dried completely in a speed vac. The next day, clean peptides were resuspended in 100 mM TEAB and micro-BCA peptide quantification was performed to obtain the amounts of peptides for each sample for subsequent labeling with 16 isobaric plexes of TMT reagent. Amine reactive TMT molecules can modify the N-terminus and side chains of lysines and have been phenomenal in performing tandem mass spectrometry by multiplexing multiple samples. An equal amount of each peptide sample was incubated with individual TMT plex reagents according to the manufacturer’s instructions (Thermo Fisher Scientific). After incubating for 60 min at RT, the reaction was stopped with 0.3% (v/v) hydroxylamine. An equal amount of isobaric labeled peptide samples were combined 1:1:1:1:1:1:1:1:1:1:1:1:1:1:1:1 and subsequently desalted with C18 HyperSep columns. The combined isobaric-labeled peptide solution was fractionated into eight fractions per manufacturer’s instructions using high pH reversed-phase peptide fractionation columns (Thermo Fisher Scientific, Cat# PI84868). Collected fractions were dried in a speed vac, and stored at − 80 °C.

### Statistical analysis

Statistical analyses were conducted using GraphPad Prism, v9. All values in figures with error bars are presented as mean ± standard error of the mean (SEM). Comparison between groups was performed using unpaired Student’s t-tests or one-way ANOVA with post-hoc Sidek test and *p*-values calculated; *p* < 0.05 were considered statistically significant. Multiple test correction was performed with the Benjamini–Hochberg correction.

## Results

### Development of a biochemical purification scheme to isolate amyloid fibrils from brain extracts

**Fig. 1:**
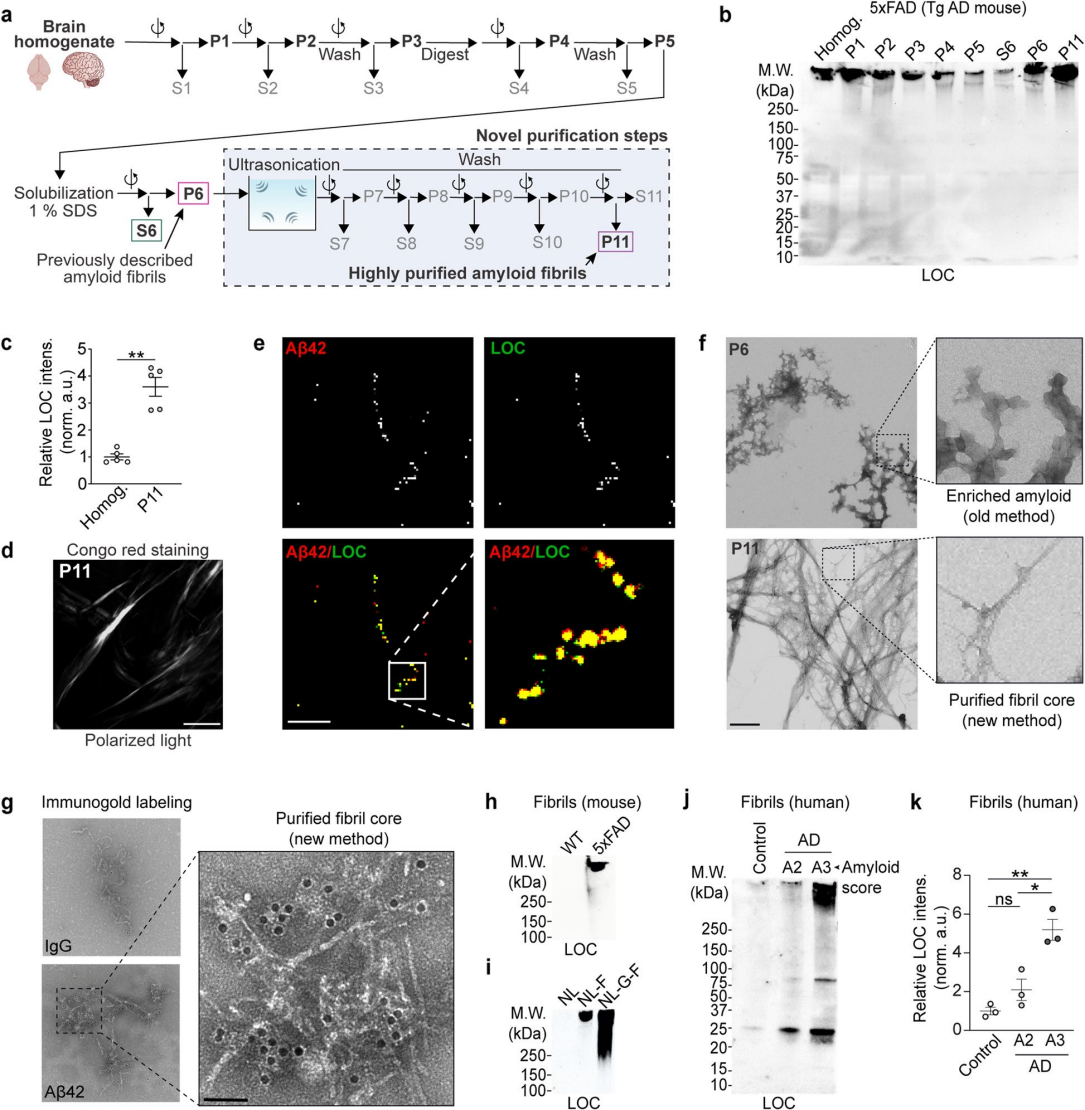
A purification strategy to isolate amyloid fibrils from AD and AD model brain extracts. **a** Biochemical purification strategy schematic. This method builds on previously developed methods and purifies SDS-insoluble dense amyloid fibril cores with sucrose density-gradient ultracentrifugation, ultrasonication, and washing with SDS. P = pellet, and S = supernatant. P6 = previously reported amyloid fibrils, P11 = highly purified amyloid fibrils. **b** Western blot analysis of indicated fractions collected during amyloid fibril purification from transgenic 5xFAD cortical extracts using anti-fibril (LOC) antibody. 10% v/v material from each fraction was loaded. **c** Normalized relative abundance of LOC-positive species in P11 with respect to input (brain homogenate). **d** Representative purified amyloid material (i.e., P11 fraction from *App*^*NL−G−F/NL−G−F*^ cortical extracts) stained with Congo red (CR) and visualized under cross polarized light. The image was captured using a monochromatic camera and is presented in greyscale. **e** Immunofluorescence (IF) images of P11 fractions from *App*^*NL−G−F/NL−G−F*^ mouse using Aβ42 and LOC antibodies. **f** Representative negative staining EM analysis of amyloid material (P11) extracted using our purification strategy from *App*^*NL−G−F/NL−G−F*^ brains compared to amyloids (P6) enriched using previously reported purification strategy. **g** Immunogold labeling with Aβ42 antibodies of purified fibrils, visualized by negative staining EM. IgG antibody was used as negative control. **h**, **i** WB analysis of purified fibrils isolated from cortical extracts of WT, 5xFAD, and *App KI* (*App*^*NL/NL*^, *App*^*NL−F/NL−F*^, and *App*^*NL−G−F/NL−G−F*^) mouse lines with contrasting levels of amyloid pathology. **j**, **k** WB analysis and quantification of amyloid fibrils isolated from human brain tissues with increasing amyloid pathology (amyloid scores) with LOC antibody. Data in c and k represents mean ± SEM; *, *p*-value < .05; **, *p*-value < .01; ***, *p*-value < .001; analyzed with unpaired Student’s t-test or one-way ANOVA with post-hoc Sidek test. NL = *App*^*NL/NL*^, NL-F = *App*^*NL−F/NL−F*^, NL-G-F = *App*.^*NL−G−F/NL−G−F*^; P = pellet, and S = supernatant. *N* = 3 mice (**d**, **e**, and **g**), 5 mice (**b**, **c**), 6—8 mice (**h**, **i**), 5 mice (**f**); *N* = 3 (**j** and **k**). All mice were 6 months of age. Scale bar = 100 µm (**d**), 10 µm (**e**), 500 nm (**f**), 50 nm (**g**)

We developed a biochemical purification strategy based on sucrose-density gradient centrifugation and ultrasonication to isolate amyloid fibrils from amyloid mouse models and post-mortem AD brains (Fig. 1a) [29]. Ultrasonication provides shearing forces sufficient to dissociate the large amyloid aggregates into SDS-resistant fibrils (Fig. S1a-b). As a pilot, we used 5xFAD transgenic brains, which display a diverse collection of amyloid plaques to assess the recovery and enrichment of the fibrils with LOC and Aβ42 antibodies. Examination of the biochemical fractions across our purification and densitometry-based quantification of high molecular weight (HMW) species revealed that the final material (i.e., P11) was highly enriched with Aβ42-containing fibrillar species (Figs. 1b-c and S1c). To extend our method using a more physiologically relevant model of amyloid-related pathology, we repeated these experiments using *App* knock-in (KI) mouse models containing humanized Aβ peptide amino acid sequence along with the Swedish mutation (*App*^*NL/NL*^), in combination with the Beyreuther/Iberian mutation (*App*^*NL−F/NL−F*^) and the Arctic mutation (*App*^*NL−G−F/NL−G−F*^) [26, 32].

To verify the purified protein aggregates contained amyloid, we stained the material with the amyloid-specific diazo dye Congo red (Figs. 1d and S1d). In parallel, an Aβ42 antibody confirmed that the purified amyloid fibrils were loaded with Aβ42 peptides (Fig. 1e). To coarsely assess the structural diversity of the purified material, we performed negative staining electron microscopy (EM), which revealed the presence of SDS-resistant individual amyloid fibrils and fibril bundles (Figs. 1f and S1e). These fibrils contained Aβ_1-42_ based on immunogold labeling (Figs. 1g and S1f). We found that 5xFAD, *App*^*NL−F/NL−F*^, and *App*^*NL−G−F/NL−G−F*^, but not wild type or *App*^*NL/NL*^ brains harbor fibrils (Fig. 1h-i). Filter trap dot blot analysis with LOC (fibrils), A11 (Aβ oligomers), and 6E10 (Aβ_1-16_) antibodies also revealed the presence of amyloid fibrils (Fig. S1g). Next, we quantified the insoluble Aβ peptides (without GuHCl solubilization) with solid-phase sandwich ELISA. The results indicate significantly higher Aβ aggregates in all three: *App*^*NL−F/NL−F*^, *App*^*NL−G−F/NL−G−F*^ and 5xFAD brains at six months compared to age matched *App*^*NL/NL*^ brains (Fig. S1h). To test the specificity of our strategy for purifying HMW fibrillar assemblies, we isolated amyloid fibrils from *App* KI mouse brain extracts at ages with increasing degrees of amyloid pathology. Notably, a progressive deposition was observed in an age-dependent manner consistent with previous reports (Fig. S1i). We extended this strategy to postmortem sporadic AD human brain tissues with increasing degree of amyloid pathology. The individual AD patient brains used were grouped based on their amyloid spread, Braak and CERAD (ABC) scores (Fig. S1j, see Table S1 for patient details) [28, 37, 38]. We also included healthy control human brains as negative controls for all experiments. First, we applied our amyloid purification strategy to isolate fibril cores from the cohort of postmortem human control and AD brain extracts. WB and ELISA revealed a significantly increased abundance of HMW aggregates in insoluble amyloids purified from the human AD brain extracts compared to control samples (Figs. 1j-k and S1k-l). Based on the results from multiple assays, we have developed a robust biochemical purification strategy to isolate amyloid fibrils.

### Aβ38 is present in amyloid fibrils

**Fig. 2:**
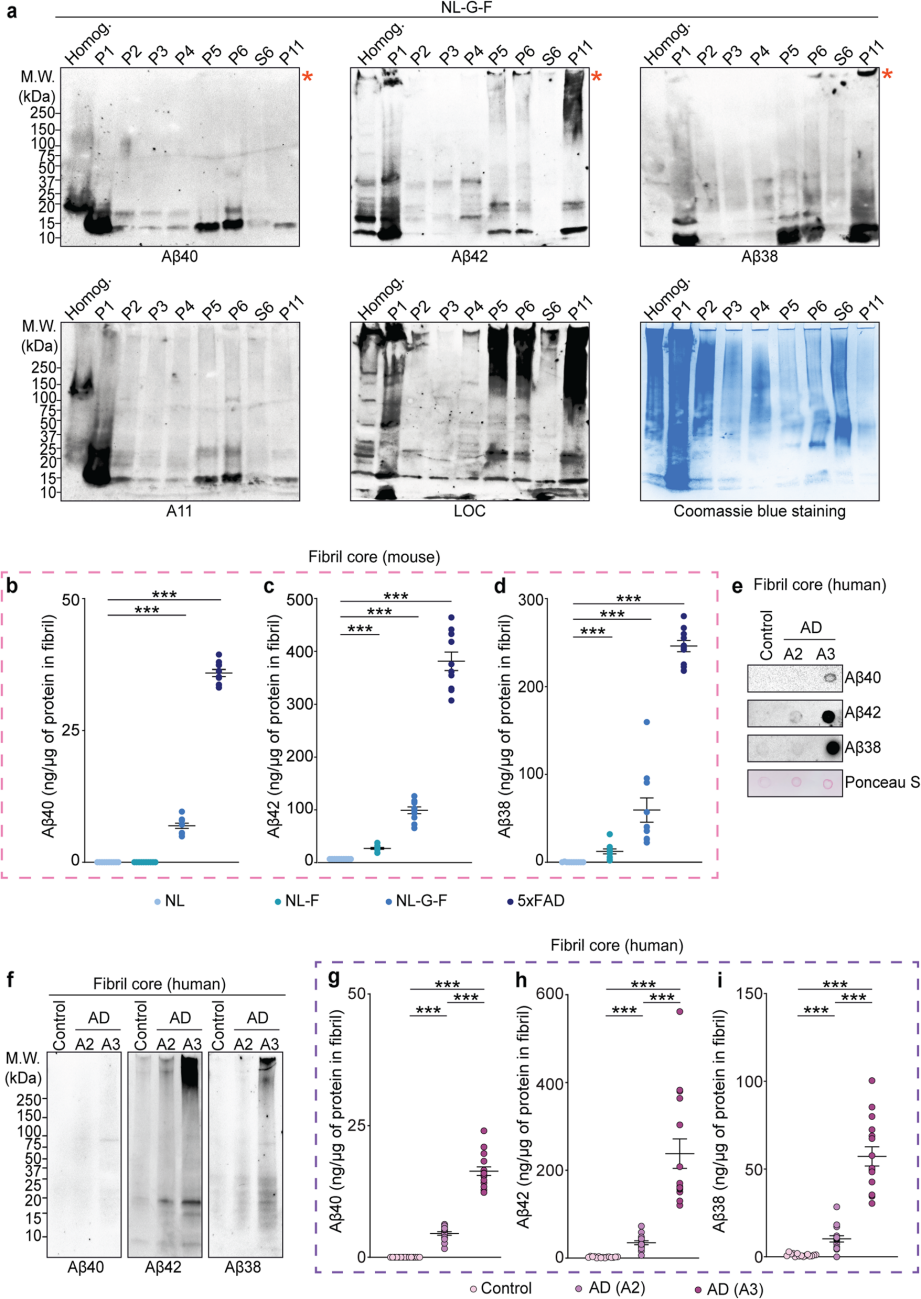
Amyloid fibril cores are enriched with Aβ42 and Aβ38 peptides. **a** WB analysis across indicated fractions from *App*^*NL−G−F/NL−G−F*^ mouse cortical extracts with Aβ40, Aβ42, and Aβ38 specific antibodies. A11, LOC blots, and Coomassie brilliant blue staining indicate abundance of oligomers, fibrils and total protein, respectively. Red asterisks indicate HMW aggregates. **b**-**d** Absolute quantification of Aβ40, Aβ42 and Aβ38 peptides in purified SDS-resistant amyloid fibrils from cortical extracts of *App KI* (*App*^*NL/NL*^, *App*^*NL−F/NL−F*^, and *App*^*NL−G−F/NL−G−F*^) and 5xFAD mice using sandwich ELISAs. **e**, **f** Dot blot and WB analysis of fibril cores obtained from postmortem AD brain tissues using antibodies for Aβ40, Aβ42 and Aβ38. Ponceau S-stained membrane in (e) was used for visualization of loading protein amount. **g**-**i** Absolute quantification of Aβ40, Aβ42 and Aβ38 peptides in purified fibrils from AD human brains. Fifteen amyloid samples from each indicated group were analyzed. Data in b-d and g-i are mean ± SEM; *, *p*-value < .05; **, *p*-value < .01; ***, *p*-value < .001; analyzed with unpaired Student’s t-test or one-way ANOVA with post hoc Sidak test. P = pellet, and S = supernatant. NL = *App*^*NL/NL*^, NL-F = *App*^*NL−F/NL−F*^, NL-G-F = *App*.^*NL−G−F/NL−G−F*^. *N* = 3 mice (**a**), 10 (**b**-**d**); *N* = 3 humans (**e**–**f**), 15 (**g**-**i**)

Aβ peptides are produced in several lengths. Thus, we purified amyloid fibrils from *App*^*NL−G−F/NL−G−F*^ brain extracts and the presence of the three most common Aβ isoforms (Aβ38, Aβ40, and Aβ42) were investigated by multiple antibody-based assays. To study the relative abundance and distribution of these three Aβ species, we collected intermediate fractions during amyloid fibril purification from *App*^*NL−G−F/NL−G−F*^ mouse brain. First, we confirmed the specificity of all three Aβ antibodies by immunoblotting recombinant human Aβ38, Aβ40, and Aβ42 peptides, respectively (Fig. S2a-c). Next, we studied the presence of individual Aβ peptide species and assemblies across the biochemical fractions using WB and filter trap dot blots (Figs. 2a and S2d). While these confirmation specific antibodies have been widely used it is important to acknowledge that it is unlikely that they can recognize all amyloid structures with the same affinity. Purified fibrils predominantly contained Aβ38, and Aβ42, while Aβ40 was the least abundant (Fig. 2a). ELISA analysis of the guanidine-solubilized material confirmed that all three Aβ peptides were significantly enriched in the purified material isolated from the 5xFAD, *App*^*NL−F/NL−F*^ and *App*^*NL−G−F/NL−G−F*^ mouse brains (Fig. 2b-d). Furthermore, amyloid fibril cores isolated from human AD brains also contained all three Aβ peptides (Fig. 2e-f). Finally, Aβ ELISA analysis confirmed that all three Aβ peptides were enriched in the purified fibrils from AD human brains (Fig. 2g-i).

**Fig. 3:**
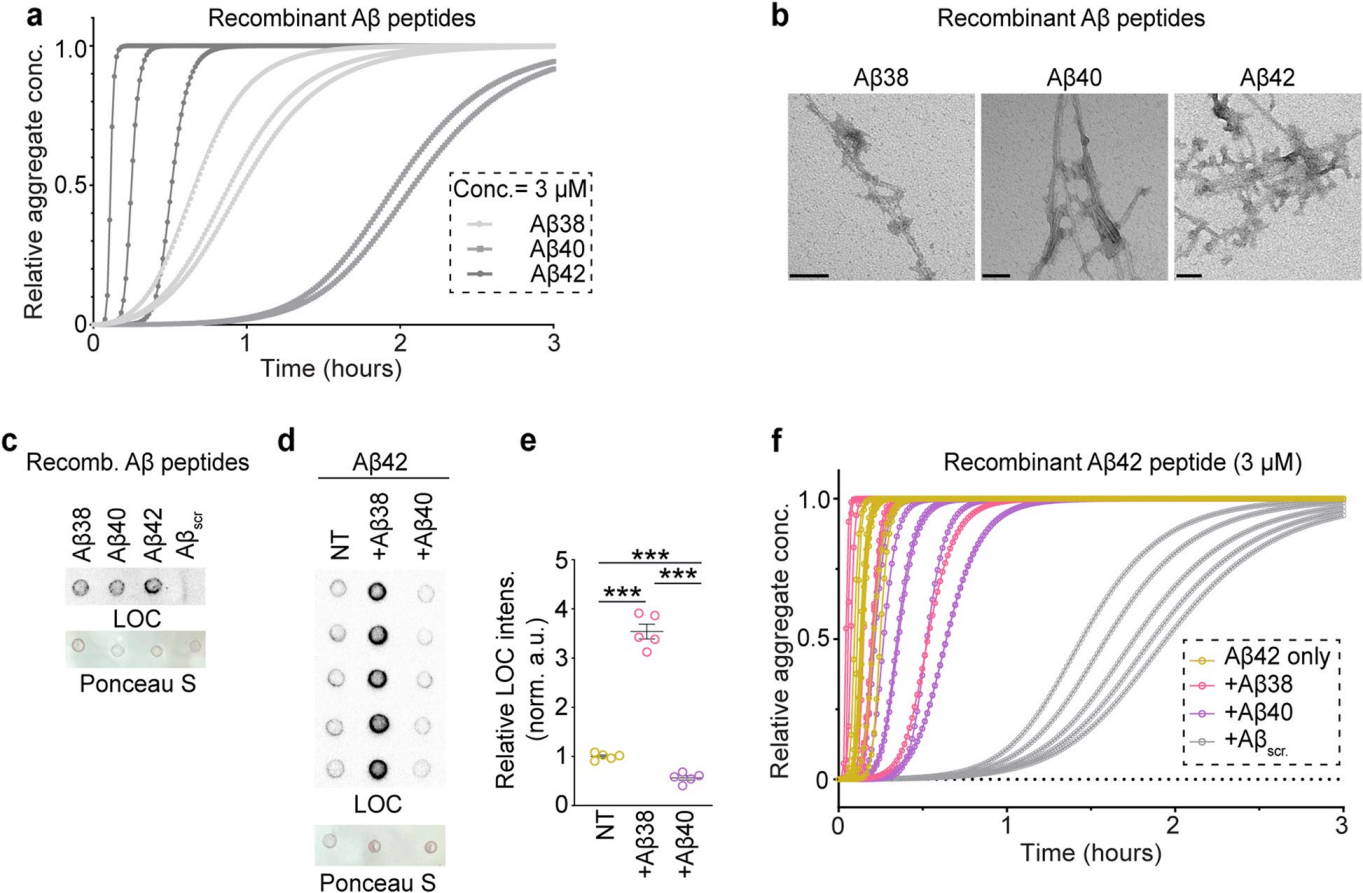
Aβ38 can seed fibril formation in vitro. **a** ThT amyloid binding dye-based aggregation kinetics of 3 µM solutions of recombinant Aβ38, Aβ40, and Aβ42 peptides prepared in aggregation buffer with 20 µM ThT. Monomeric peptide solutions were prepared using GuHCl solubilization prior to setting up the experiments. **b** Representative negative staining electron microscopy images of recombinant Aβ38, Aβ40, and Aβ42 peptides incubated for 72 h at room temperature. **c** Dot blot analysis using LOC antibody reveals relative levels of inherent fibrils formed in vitro from recombinant Aβ38, Aβ40 and Aβ42 peptides following GuHCl solubilization*.* Scramble* A*β42 peptides were used as negative control. Ponceau S-stained membranes were used for visualization of loading protein amount. **d**, **e** Dot blot analysis using LOC antibody for peptides obtained from 24 h incubation of 3 µM monomeric Aβ42 alone or with other monomeric peptides incubated at 100 nM concentration. Ponceau S-stained membranes were used for visualization of loading protein amount. **f** ThT-based amyloid kinetics of two-peptide system consisting of 3 µM Aβ42 in absence or presence of other peptides (Aβ38, Aβ40 and scramble Aβ42) at 100 nM concentration. The relative amyloid concentrations were calculated using secondary nucleation model in AmyloFit online tool (https://amylofit.com/amylofitmain). ThT fluorescence intensities were measured every four minutes. *N* = 3 replicates (**a**, **c**), 5 (**d**-**f**). Scale bar = 50 nm (**b**)

To investigate the contribution of Aβ38 and Aβ40 peptides to amyloid fibril formation, we performed in vitro experiments using the ThT-based kinetic assay, dot blots, and EM analysis (Fig. 3a-c). Next, we performed two peptide analyses to examine the effect of 100 nM Aβ38 or Aβ40 on the aggregation kinetics of 3 µM Aβ42 peptides. Notably, the presence of either two peptides (100 nM Aβ38 or Aβ40), influenced the rate of Aβ42 aggregation (Fig. 3d-f). Consistent with previous reports, we found that the presence of Aβ42 enhances Aβ40 aggregation (Fig. S3b). Notably, Aβ42 had no effect on Aβ38 aggregation (Fig. S3c). In summary, these results indicate that SDS-resistant amyloid fibril cores are formed primarily of Aβ42 and some Aβ38; and Aβ38 can positively influence Aβ42 fibril formation in vitro.

### Multiscale profiling of the amyloid fibril proteome

**Fig. 4:**
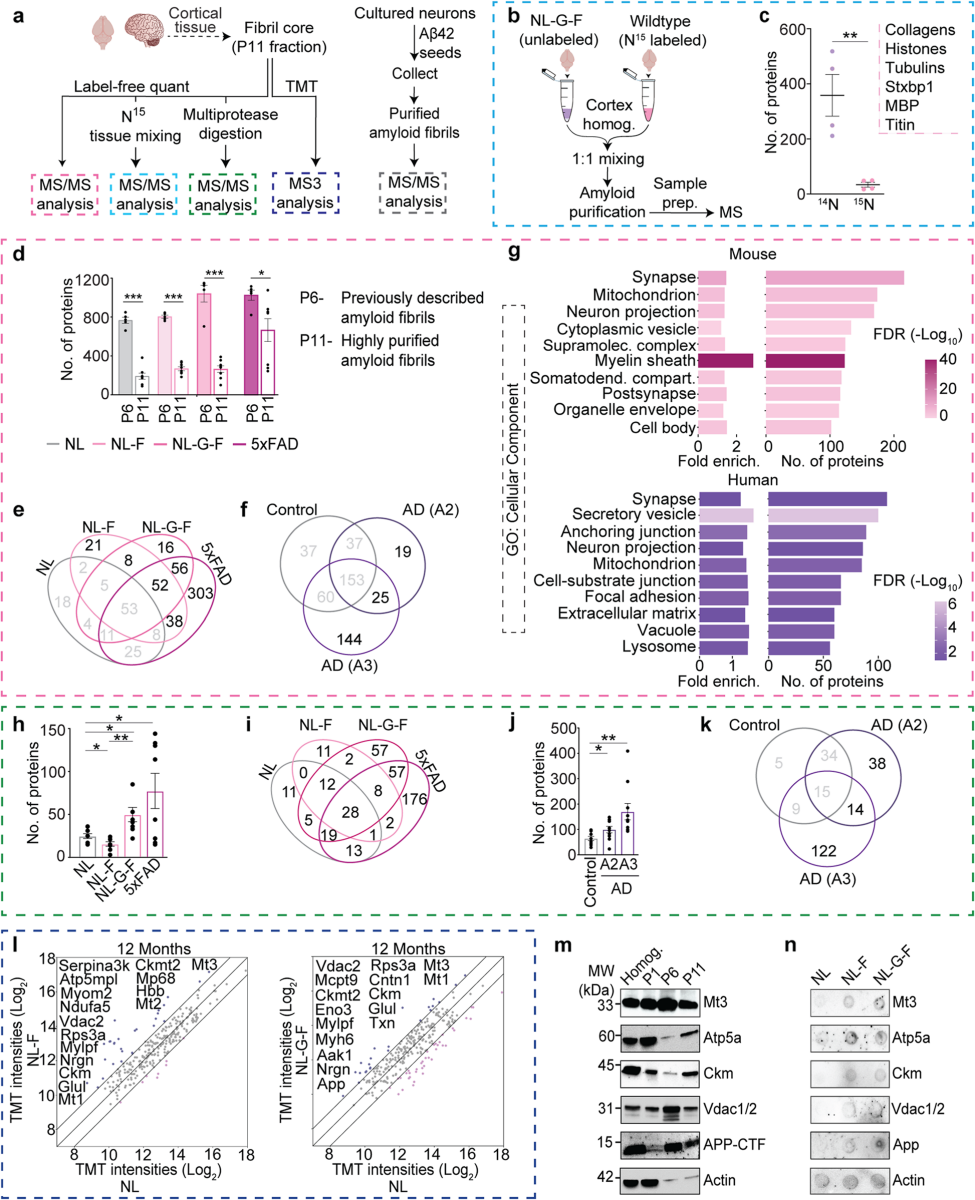
Multidimensional MS-based proteomic analyses identify amyloid fibril proteome. **a** Summary of MS analyses performed. **b** Experimental workflow using ^15^N-labeled brain tissue as a control to identify nonspecific co-purifying proteins. **c** Only a small panel of non-specific background proteins are identified in purified amyloid fibrils based on the identification of ^14^N and ^15^N labeled proteins; small inset shows identified ^15^N proteins. **d** The new purification strategy significantly reduces the number of proteins identified in P11 fractions compared to P6 fractions collected from *App* KI (*App*^*NL/NL*^, *App*^*NL−F/NL−F*^, and *App*^*NL−G−F/NL−G−F*^) and transgenic 5xFAD brains. **e** Venn diagram depicting the number of proteins identified in purified fibril cores across the indicated mouse strains at a 1% protein FDR. **f** Venn diagram comparing proteins identified in fibrils isolated from control and AD human brains. **g** The GO-cellular components analysis with proteins identified in mouse (**e**) and human (**f**) amyloid fibrils. **h** Number of proteins identified in label-free MS analysis of amyloid fibril cores extracted from mouse brain tissues following digestion with multiple proteases. **i** Venn diagram comparing number of proteins identified across different mouse strains in multi-protease digestion (h) LC–MS/MS analysis. **j** Number of proteins identified in label-free MS analysis of fibril cores following multiprotease digestion of human brain-derived amyloid fibrils. **k** Venn diagram comparing number of proteins identified across human fibrils (**j**) digested with multiple proteases. **l** Scatter plots comparing average TMT intensities of *App*^*NL−F/NL−F*^, and *App*^*NL−G−F/NL−G−F*^ with *App*^*NL/NL*^ fibril cores. Two biological replicates were pooled for each TMT channel. **m** Representative immunoblots confirming the presence of selected proteins identified in the proteomic analyses. **n** Representative dot blots for same proteins in *AppKI* amyloid fibrils. Data in **c**, **d**, **h**, and **j** represents mean ± SEM; *, *p*-value < 0.05; **, *p*-value < 0.01; ***, *p*-value < 0.001; ****, *p*-value < 0.0001 analyzed with unpaired Student’s t-test or one-way ANOVA with post hoc Sidak test. P = pellet, and S = supernatant. *NL* = *App*^*NL/NL*^, *NL-F* = *App*^*NL−F/NL−F*^, *NL-G-F* = *App*^*NL−G−F/NL−G−F*^. All mice were 6 months of age unless indicated. *N* = 4 mice (**c**), 4—8 mice (**d** and **e**), *N* = 15 control, 13 AD A2, 23 AD A3 humans (**f**), 7–8 mice (**h**, **i**), 10 humans (**j**, **k**)

To identify proteins involved with the formation or stabilization of amyloid fibrils, we analyzed the purified material with MS-based proteomic analysis using five complementary workflows (Fig. 4a). To ensure that the proteins identified by MS are truly associated with fibril cores rather than representing co-purifying impurities, first we mixed *App*^*NL−G−F/NL−G−F*^ brain homogenates with WT brains metabolically labeled with ^15^N Spirulina chow. In this way, any ^15^N protein identified must have associated in the tube during purification and was thus deemed non-specific (Fig. 4b). MS analysis revealed that > 90% of the proteins identified were ^14^N-labeled, while the remaining 10% were ^15^N-labeled (e.g., collagen, histones, titin, tubulin, myelin basic proteins and syntaxin-binding protein 1) and no longer considered as being present in the fibril cores (Fig. 4c). Next, we confirmed that our modified purification strategy resulted in significantly reduced number of non-specific co-purifying proteins and increased the purity. In all four mouse models, we significantly reduced the number of identified proteins compared to material prepared using previous purification method (Fig. 4d). By comparing the proteins identified in the material isolated from *App*^*NL−F/NL−F*^, *App*^*NL−G−F/NL−G−F*^, and 5xFAD brains, relative to control *App*^*NL/NL*^ brains, we delineated the proteins associated with pathological forms of amyloid and later highlighted proteins identified in multiple models (Fig. 4e). However, the relationship between the number of proteins identified could be a reflection of the number of plaques or the number of proteins present in each plaque, unfortunately we have no way of knowing. Notably, the ELISA results suggest there is increasingly more Aβ42 in the purified material from 5xFAD > *App*^*NL−G−F/NL−G−F*^ > *App*^*NL−F/NL−F*^ > *App*^*NL/NL*^*.*

In the fibril cores isolated from human brain tissues, we identified the largest number of proteins from fibril samples prepared from A3 brains, followed by A2 brains (Fig. 4f), and it is possible that this result is influenced by the overall number of plaques present. Next, we compared the relative abundance of proteins in the purified material relative to the starting material (i.e., cortical homogenate). Most proteins were identified in both analyses, with small fraction of proteins being enriched by 20-fold or more in mouse or human extracts (Fig. S4a-b, Tables S2and S3). A panel of the most significantly enriched proteins were present in the material purified from multiple mouse models and amyloid stage human brains (Fig. S4c-f). Gene Ontology (GO) for cellular component (CC) enrichment analysis revealed many of the fibril-associated proteins are associated with synapse, neuron projection, myelin sheath, supramolecular complex, and extracellular matrix (Fig. 4g). To complement these studies, we also subjected the amyloid fibrils to multiple proteases to remove the proteins on the fibril periphery and to liberate peptides tightly associated with the inner fibril core (Fig. S4g). We identified significantly more proteins in the *App*^*NL−F/NL−F*^, *App*^*NL−G−F/NL−G−F*^ and 5xFAD fibrils compared to *App*^*NL/NL*^ (Fig. 4h-i and Table S4). In human samples, we identified the most proteins in the purified material from Amyloid score 3 brains followed by those with A score 2 (Fig. 4j-k and Table S4).

To obtain more rigorous quantification of the individual proteins in fibrils from all three *App* KI mouse lines at 12 and 18 months of age, we performed a 16-plex TMT experiment (Fig. S4h). We used WT (C57BL/6) cortical and *App*^*NL−G−F/NL−G−F*^ cerebellar extracts as controls for these experiments. The biological replicates were clustered in PCA analysis based on the genotype and age (Fig. S4i). Mt1, Mt3, Ckm, and Vdac2 were present at levels at least twofold greater in both *App*^*NL−F/NL−F*^ and *App*^*NL−G−F/NL−G−F*^ compared to *App*^*NL/NL*^ fibrils isolated from 12-month-old mice (Fig. 4l). In fibrils from 18-month-old mice, mitochondrial protein (Hadha), and the cytosolic malate dehydrogenase (Mdh1) met the same criteria (Fig. S4j). Notably, Mt3 stood out as a top candidate since it was present at levels greater than twofold in fibrils from *App*^*NL−G−F/NL−G−F*^ cortex compared to the cerebellum, *App*^*NL−G−F/NL−G−F*^ compared to *App*^*NL−F/NL−F*^ at 18 months, and finally *App*^*NL−G−F/NL−G−F*^ at 18 months compared to 12 months (Fig. S4k-m). By comparing TMT intensities of proteins identified in fibrils, we first identified proteins that were two-fold enriched in fibrils from *App*^*NL−F/NL−F*^ and *App*^*NL−G−F/NL−G−F*^ amyloids, as compared to *App*^*NL/NL*^ at 12 and 18 months of age (Figs. 4l, S4j and Table S5). We also homed in on proteins, which were selectively enriched in fibrils from cortex compared to those purified from the cerebellum of eighteen-month-old *App*^*NL−G−F/NL−G−F*^ mice (Fig. S4k). Moreover, we identified proteins that were selectively enriched in fibrils from *App*^*NL−F/NL−F*^ with mild amyloid pathology compared to cortical fibrils from an aggressive amyloid pathology brain (*App*^*NL−G−F/NL−G−F*^) of the same age (Fig. S4l). Comparison of the protein levels from 12- and 18-month-old *App*^*NL−F/NL−F*^ and *App*^*NL−G−F/NL−G−F*^ brains revealed proteins that bind to fibrils in an age-dependent manner (Fig. S4m). Next, we validated the MS findings with a panel of antibodies and confirmed that most of these proteins are abundant in the P11 fraction (Figs. 4m-n and S4n-p).

To confirm our putative amyloid fibril proteome, we incubated rodent hippocampal and cortical neurons with recombinant Aβ42 peptides and used our purification strategy to isolate amyloid fibrils and associated proteins (Fig. S5a). As a first step, we performed Aβ42 WB and confirmed the presence of abundant HMW amyloid species (Fig. S5b). Notably, more than two-thirds of the proteins were identified in the amyloid fibrils isolated from both cortical and hippocampal neurons (Fig. S5c). Forty-nine proteins were present in both amyloid fibrils formed in vitro and in vivo (Fig. S5d and Table S6). Finally, we confirmed several proteins identified in the MS and biochemistry analyses being present in amyloid plaques by immunofluorescence (Fig. S5e-j). In summary, our multiscale proteomic analysis provided a short rank-ordered list of proteins physically associated with amyloid fibrils.

### Metallothionein-3 can affect amyloid fibril formation

To test if the proteins we discovered closely associated with the amyloid fibril can influence fibril formation, we tested one candidate protein MT3 that was prominent in the TMT and multiple protease proteomic datasets (Fig. 4l-n and Tables S4 and S5). MT3 is a small cysteine-rich protein that regulates metal ions (e.g., Cu^2+^ and Zn^2+^) and is expressed primarily in the brain [39]. MT3 levels are reduced in AD brains, but little is known about this protein’s role in amyloid pathology [40]. First, we confirmed an MT3 antibody with recombinant and brain derived MT3 proteins (Fig. S6a). Following which, immunogold labeling of amyloid fibrils with the MT3 antibody verified its presence in fibrils (Fig. S6b). Next, using dot blot analysis we observed relative MT3 protein level in *App KI* mouse brain cortex homogenates, purified fibrils and Aβ42 immunoprecipitates (Fig. S6c). Immunofluorescence analysis using Aβ42 and MT3 antibodies of purified fibrils revealed strong co-localization of MT3 protein with Aβ42 peptides (Fig. S6d). To further quantify the relative level of MT3 in amyloid fibril cores, we performed sandwich ELISA and found the amount scaled with the amount of Aβ42 peptides in amyloid fibrils (Fig. S6e-f). To investigate if MT3 can influence Aβ aggregation, we performed in vitro assays with recombinant Aβ38, Aβ40, and Aβ42 peptides. To investigate if presence of MT3 protein affects amyloid formation, we performed ThT-based kinetic assays. We found that the presence of MT3 protein increased the lag time (i.e., slowed the initiation of aggregation) but has no impact on the overall extent of aggregation (Fig. S6g).

### Proteins associated with the amyloid fibril core modify amyloid toxicity in vivo

**Fig. 5:**
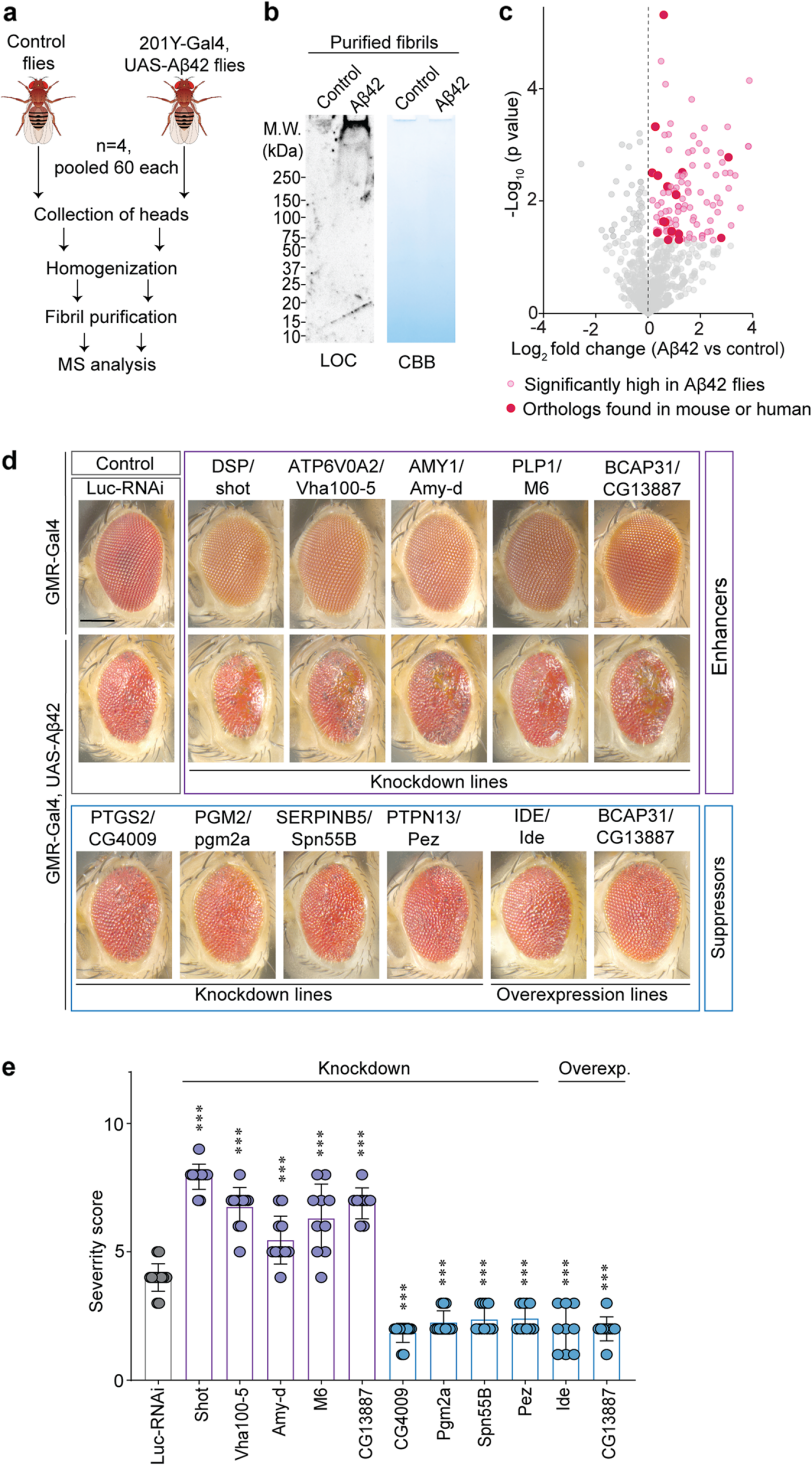
Mouse and human fibril protein orthologs interact with Aβ42 peptides in vivo and modulate amyloid toxicity in *Drosophila*. **a** Biochemical purification workflow and proteomic analysis of amyloid fibril core from transgenic flies expressing Aβ42 in adult brain using the 201Y-Gal4 driver. **b** Representative WB analysis of the purified material isolated from *LacZ* control and Aβ42 flies using LOC antibody. **c** Volcano plot depicting relative abundance of proteins in fibrils isolated from flies expressing Aβ42 in adult brain compared to innocuous *LacZ* control flies. **d** Representative eye images of Aβ42-expressing flies carrying the indicated RNAi or overexpression lines for shortlisted genes from the MS analysis. Note that the enhancers do not modify the eye morphology in the absence of Aβ42. Luc-RNAi and UAS-*LacZ* were used as negative controls against RNAi and overexpression lines, respectively. **e** Histograms represent the severity scores of the indicated genetic modifiers compared to control flies. Data in e represents mean ± SD; **, *p*-value < .01; ***, *p*-value < .001; analyzed with ordinary one-way ANOVA followed by Dunnett’s multiple comparison test. *N* = 4 biological replicates, 60 flies pooled in each sample (**c**), 8–15 flies per line (**e**). Scale bar = 100 µm (**d**)

To assess the functional impact of the amyloid fibril-associated proteins on amyloid-induced toxicity, we used a well-established *Drosophila* model of Aβ42 deposition [33]. In support of this model system, a recent study confirmed that Aβ42 forms fibrils and induces neurotoxicity in fly brains [41]. We first tested if Aβ42 peptides form fibrils in neurons of the fly brain. For this, we collected heads from flies expressing Aβ42 in the Kenyon cells of the mushroom bodies (linked to learning and memory) using 201Y-Gal4 driver. We then purified fibrils using our newly described method (Fig. 5a). WB analysis with LOC antibodies confirmed the presence of HMW amyloid fibrils in the heads of Aβ42-expressing flies (Fig. 5b). MS-based proteomic analysis of isolated fibrils revealed 169 proteins with significantly higher levels compared to WT controls (Figs. 5c and S7a). Additionally, 110 proteins identified in the fly amyloid fibrils are orthologs to the mammalian (25 human and 85 mouse) fibril-associated proteins (Table S7). Taken together, while not equivalent to the mammalian systems, the fly model displays similar biology in fibril formation and serves as a useful tool.

Next, we determined if modulating the expression of fly genes, orthologous to genes encoding proteins found in mouse amyloid fibrils, can modify Aβ42-induced toxicity in the fly eye. For this, we capitalized on the robust Aβ42 eye phenotype induced upon expression with the eye-specific GMR-Gal4 driver. This phenotype has 100% penetrance and is a highly reliable platform to test genetic modifiers of Aβ42-mediated toxicity [34]. We tested 60 RNAi or overexpression lines corresponding to those mouse gene orthologs identified in amyloid fibril cores. Seven RNAi lines suppressed and nine RNAi lines enhanced Aβ42 toxicity (Figs. 5d-e, S7b-c and Table S7). Notably, *CG4009*, *pgm2a*, *spn55b*, and *pez* (orthologues to mouse *Prkdc*, *Pgm2*, *Serpinb5*, and *Ptpn13* genes, respectively) showed a prominent rescue of Aβ42 insults. A small panel of fly lines over-expressing fly orthologs or human genes encoding amyloid fibril-associated proteins were obtained and crossed with GMR-Gal4 > Aβ42 flies. Among these, we found two fly genes that suppress (*Ide* and *Bcap13*) and three human genes (*SCAMP5*, *DUSP14*, and *LOX*) that enhance Aβ42 toxicity (Figs. 5d-e, S7d and Table S7).

## Discussion

We set out to investigate how amyloid fibrils are formed and stabilized by mapping the amyloid fibril proteome in three model systems (mice, cultured neurons, flies) and human AD brain. Our customized biochemical purification strategy is designed specifically to isolate dense SDS-insoluble aggregates. By incorporating ultrasonication-based vibrational disruption of weakly associated proteins, combined with extensive washing, we significantly reduced the number of copurifying proteins by two-to-five-fold (Fig. 4d). However, these shearing forces were not sufficiently strong to break covalent bonds or remove tightly associated binding proteins. Stable isotope labeled mouse brains served as a reliable internal standard allowing us to systematically determine the proteins that nonspecifically copurify with amyloid fibrils (Fig. 4b-c). To validate our proteomic results, we confirmed the presence of the identified proteins by immunoblots and immunohistochemistry of three months-old *App*^*NL−G−F/NL−G−F*^ mouse brain sections. We have thus overcome the inconsistencies previously encountered in large-scale proteomic studies and isolated fibrils with little to no contamination, which allows us to identify biologically relevant proteins, associated with amyloid fibril cores.

Previous MS-based studies have shown a sequential cleavage of APP by γ-secretase leading to generation of Aβ peptide fragments from 30 to 51 amino acids [42]. The shorter peptides (e.g., Aβ38 or Aβ40) are historically considered less-toxic and unable to cause behavioral deficits in fly and mouse models [5]. In fact, application of γ-secretase modulators (GSMs) leads to a decrease in larger Aβ peptides (i.e., 42 and 43) that are both substrates and products of γ-secretase in mice [43, 44]. Several previous and ongoing studies have targeted γ-secretase activity as an anti-AD therapeutic strategy with some success [45, 46]. For example, a recent pharmacological study using pyridazine-based GSMs found reduced net production of Aβ42 and to a lesser degree Aβ40, while concomitantly enhancing production of Aβ38 and Aβ37 [47]. Furthermore, this GSM could reduce the amyloid plaque load in double transgenic mice. At face value, this could be at odds with our findings based on the correlation between reduced plaques and elevated Aβ38 levels; however it is also possible that the effect was due solely to reducing the level of Aβ42. Other evidence indicates production of Aβ38 in Aβ42-independent manner, contradicting precursor-product relationship among the two and abolishing effects of multiple GSMs [48, 49]. Importantly, Aβ38 peptides have been detected at extracellular amyloid plaques in sporadic and familial AD patients and mouse models [50]. Additionally, recent MS-based imaging of *App*^*NL−G−F/NL−G−F*^ brains showed that Aβ38 is deposited specifically during plaque growth and may crosstalk with Aβ42 peptides [51]. Similarly, there are discrepancies regarding the abundance of Aβ40 in amyloid plaques. For example, Iwatsubo et al. confirmed that both senile and diffuse plaques are primarily composed of Aβ42, and lack Aβ40 [52]. Another study by Upadhaya et al. showed that Aβ40 peptides mostly form SDS-soluble oligomeric and protofibrils, while Aβ42 is the major constituent of SDS resistant HMW fibrils [53]. It is important to note that in a previous biochemical analysis of purified plaque-derived 7 kDa—Aβ fractions from AD brains Aβ40 was detected in all five of the brains analyzed, while Aβ38 was detected in only three out of the five brains analyzed [54]. Therefore, we acknowledge that our results are not completely consistent with these previous findings. Additionally, an in vitro analysis showed that shorter Aβ peptides (Aβ37, Aβ38, and Aβ40) can modulate Aβ42 fibril formation at high concentrations [55]. Another study performed in AD patients reported a lower risk of AD-related changes in patients with high CSF Aβ38 levels [56].

In our studies, we found Aβ38 peptides are highly abundant in SDS-resistant amyloid fibrils purified from mouse and AD human brains. More importantly, using ThT based amyloid kinetic assays, we confirmed that the presence of Aβ38 peptides (~ 3% v/v solution) can modulate aggregation of Aβ42 peptides at 3 μM. However, these findings remain somewhat inconclusive as varying experimental conditions frequently yield dissimilar results in ThT based kinetic assays. For example, in a comprehensive in vitro analysis of multiple peptide solutions, presence of higher amount of Aβ38 did not show any impact on equivalent concentration of Aβ42 [55]. Notably, we also investigated if the purified fibrils possessed post-translationally modified Aβ peptides, which has previously been reported to influence their aggregation [57]. Unfortunately, we did not identify any Aβ PTMs; however this negative result needs to be considered with caution. These observations provide potential insight into APP processing and Aβ aggregation dynamics.

Most in vitro studies performed at micromolar concentration show a prompt aggregation of Aβ peptides; however, brain harbors these peptides only in nanomolar concentrations. That’s why the peptides take years to decades to deposit and form long fibrils. We hypothesize that there could possibly be other cellular proteins in the proximity of Aβ peptides that assist in the initial oligomerization and nucleation. We performed a line of experiments to comprehensively investigate the core proteome of the highly pure amyloid fibrils. Overall, seventy-seven proteins reproducibly identified in Aβ fibrils (from two or more sources) provide a unique perspective on where and how amyloid fibrils are formed and cause toxicity. Notably, fifty-seven of these proteins have previously been found to co-purify or co-localize with Aβ42 in the brain, indicating consistency between our results and several previous studies. Biochemical evidence confirming a direct physical interaction with Aβ42 peptides is lacking for most proteins (Table 1). Many of these proteins localize to the synapse, extracellular matrix, and organelle envelope, which is in line with several previous reports aimed at studying amyloid coronae [58]. The highly abundant cytoskeletal proteins actin, and dynein, have all been previously found to be associated with amyloid plaques using antibody-staining [59, 60]. Similarly, a neurofilament protein alpha-internexin deposited in Aβ-positive dystrophic neurites [61, 62].

**Table 1:** Summary of amyloid fibril associated proteins that have been previously found at amyloid plaques or with Aβ peptides

Protein name	Gene / Protein	Source	Literature	Reference
Heterogeneous ribonucleoproteins	*HNRNPA2B1, HNRNPH1*	M, H, T, D, N, F	MS	[63]
Serine proteases	*PRSS1*	M, H, T, D, N	IHC	[64]
14–3-3 proteins	*YWHAB, YWHAG, YWHAQ, YWHAZ, SFN*	M, H, T, D, N	MS, BC	[65–68]
Ribosomal large subunits	*RPL11, UBA52*	M, H, T, D, F	BC, MS	[63, 66]
Cofilin-1	*CFL1*	H, T, D, N	MS	[63]
Elongation factor 1-alpha 1	*EEF1A1*	M, T, D, N	MS	[63, 69]
Heat shock protein 90 family proteins	*HSP90AB1*	T, D, N, F	IHC, MS	[65, 70]
Heat shock protein 70 family proteins	*HSPA8, HSPA1L*	M, T, D, N	MS	[63, 66, 69]
Succinate dehydrogenase flavoprotein	*SDHA*	H, T, D, N	MS	[70]
Aconitate hydratase	*ACO2*	T, D, N	MS	[70]
Actin, cytoskeletal proteins	*ACTA1, ACTA2, ACTG2*	M, H, T	IHC, MS	[59, 66, 70]
Amyloid-beta precursor protein	*APP*	M, H, T	IHC, BC, MS	[2, 70]
Na + /K + transporting ATPase subunits	*ATP1A3*	M, T, N	IHC, MS	[66, 70, 71]
Calcium-transporting ATPase 1, ER	*ATP2A2*	T, D, N	IHC	[72]
Calmodulin-1, -2	*CALM1, CALM2*	H, T, D	IHC, BC, MS	[66, 73]
CaMKII subunits	*CAMK2A*	M, T, N	IHC, MS	[63, 70, 74]
Clathrin heavy chain 1	*CLTC*	T, D, N	MS	[66, 70]
Dihydropyrimidinase-related proteins	*CRMP1, DPYSL2, DPYSL3*	T, D, N	MS	[66]
Dynamin-1	*DNM1*	T, D, F	MS	[63, 65, 70]
Desmoplakin	*DSP*	M, H, T	MS	[75]
Dyneins, motor proteins	*DYNC1H1*	T, N, F	IHC, MS	[60, 63, 65]
Glyceraldehyde-3-phosphate dehydrogenase	*GAPDH*	T, D, N	MS	[63]
Glutamine synthetase	*GLUL*	T, D, N	MS	[70]
Guanine nucleotide-binding protein subunits	*GNAO1, GNB1, GNB2*	T, D, N	MS	[70]
Alpha-internexin	*INA*	T, D, N	IHC, MS	[61, 66, 70]
Malate dehydrogenase 2	*MDH2*	T, D, N	MS	[66]
Vesicle-fusing ATPase	*NSF*	T, D, N	MS	[70]
Pyruvate dehydrogenase E1	*PDHB*	T, D, N	MS	[70, 76]
Phosphoglycerate kinase	*PGK1*	H, T, N	MS	[63, 75]
Pyruvate kinase	*PKM*	T, D, N	MS	[70]
Peroxiredoxins	*PRDX6*	T, D, F	MS	[66]
Ribosomal small subunits	*RPS20*	H, D, F	MS	[63]
Excitatory amino acid transporter 2	*SLC1A2*	M, T, D	MS	[70]
Synaptosomal-associated protein 25	*SNAP25*	T, D, N	IHC, MS	[32, 63, 70]
Clathrin coat assembly protein AP180	*SNAP91*	H, T, N	IHC, MS	[32, 66]
Spectrin alpha chain	*SPTAN1*	T, D, F	IHC, MS	[59, 70]
Thioredoxin	*TXN*	M, H, T	MS	[77]
Polyubiquitin-B	*UBB, UBC*	M, H, T	IHC, MS	[63, 66, 78]
Ubiquitin C-terminal hydrolase L1	*UCHL1*	H, T, N	BC	[79]
Voltage-dependent anion channel 1, 2	*VDAC1, VDAC2*	M, T, D	IHC, BC, MS	[63, 80]
Vimentin	*VIM*	T, D, N	IHC, MS	[63, 65, 69, 81]

Proteins reproducibly identified in multiple proteomic analyses of amyloid fibrils isolated from brains (mouse, human, and Drosophila) or rat neurons using label-free or TMT 16-plex MS3-based quantification. Experimental evidence in this study- *M* Mouse (LFQ MS/MS), *H* Human (LFQ MS/MS), *D* Multiprotease digestion (LFQ MS/MS), *T* TMT analysis (MS3), *N* primary neurons (LFQ MS/MS), *F* Fly (LFQ MS/MS). Available literature evidence- *MS* proteomic study, *IHC* Immunohistochemistry, *Biochem* Biochemical interaction

Consistent with previous reports, we found many proteostasis-related proteins associated with amyloid fibrils, including HSP70, HSP90 chaperones, and ubiquitin proteasome system components, such as ubiquitin and UCHL1 [65, 78, 79]. We speculate that these proteins interact with Aβ peptides soon after they start misfolding or accumulating, probably inside of the cells to circumvent the proteotoxicity. The presence of these and other intracellular proteins is at odds with extracellular amyloid deposition, but consistent with previous findings on the intracellular production of Aβ peptides [82–84]. It is also possible that these molecular chaperones were extracellularly exported through non-conventional secretory mechanisms [85]. The calcium/calmodulin-dependent protein kinase II (CaMk2a), which is a known kinase responsible for APP phosphorylation, was also found in the fibrils [84, 86]. In our analysis, we found that overexpression of insulin degrading enzyme (IDE), a protease and knockdown of Serpinb5a, protease inhibitor, both could suppress Aβ-induced toxicity in *Drosophila* eye neurons. Interestingly, we found that siRNA gene knock down of Bcap31 (an ER transmembrane protein) enhanced toxicity while overexpression rescued toxicity. Consistently, knock out of Bcap31 in APP / PS1 transgenic mice increased the Aβ plaque load [87]. Metallothioneins are low molecular weight (LMW) cysteine-rich metal-stabilizing proteins that have been implicated in a wide range of functions in diverse tissues. However, the functionally distinct, brain-specific isoform Mt3 is a small 68 amino acid-long metal-binding chaperone protein that was initially discovered as a neuroinhibitory factor [39]. We confirmed its presence in fibril cores, while the levels in brain homogenates were almost undetectable in our dot blot analysis. The presence of recombinant Mt3 protein slows amyloid aggregation kinetics based on ThT based assay.

We identified twenty proteins that have never been found in amyloid fibrils or plaques (Table 2). Notably, reducing ACAT1 can inhibit Aβ production in AD mouse models [88]. On the other hand, peptidyl-prolyl cis–trans isomerase A (PPIA), a blood brain barrier regulatory protein, confers protective effects against Aβ-induced toxicity [89]. The mitochondrial enzyme isocitrate dehydrogenase (IDH3B) has altered expression in postmortem AD subjects compared to healthy controls [90]. In addition, we found the ADP ribosylation factor ARF5 (ER trafficking GTPases) associated with amyloid fibrils. ARF5 has never before been implicated in AD; however, ARF6, a paralog, plays an important role in APP cleavage by affecting BACE1 endosomal sorting [91]. Splicing factor SRSF4 is associated with frontotemporal dementia and Huntington’s disease and may have potential roles in AD pathology through tau [92, 93]. These observations prove that our findings are relevant to AD etiology and pathology.

**Table 2:** Newly discovered proteins found associated with purified Aβ fibrils

Protein name	Gene / Protein	Source
Acetyl-CoA acetyltransferase, mitochondrial	*ACAT1*	T, D, N
ADP-ribosylation factor 5	*ARF3, ARF5*	H, T, D
Cytochrome c oxidase subunit 6A1, mitochondrial	*COX6A1*	M, D, F
Nidogen-1	*NID1*	M, H, D
Aspartate aminotransferase, mitochondrial	*GOT2*	H, T, D
Basement membrane-specific heparan sulfate proteoglycan core protein	*HSPG2*	M, H, D
Serine peptidase	*HTRA1*	M, H, D
Isocitrate dehydrogenase [NAD] subunit, mitochondrial	*IDH3B*	T, D, F
Laminin subunit- alpha-5, gamma-1	*LAMA5, LAMC1*	M, H, D, F
Neural cell adhesion molecule 1	*NCAM1*	H, T, D
NADH dehydrogenase [ubiquinone] 1 alpha subcomplex subunit 9, mitochondrial	*NDUFA9*	H, T, D
Peptidyl-prolyl cis–trans isomerase A	*PPIA*	T, D, N
Ras-related protein Rac1	*RAC1*	H, T, D
Serine/arginine-rich splicing factor 4	*SRSF2*	M, H, D
Synapsin-1	*SYN1*	H, T, D
Thy-1 membrane glycoprotein	*THY1*	M, T, D
Tubulointerstitial nephritis antigen-like	*TINAGL1*	M, H, D
Tropomyosin alpha-3 chain	*TPM3*	H, T, D

Summary of proteins identified in our analyses that have not yet been reported to be associated with Aβ fibrils. Experimental evidence in this study- *M* Mouse (LFQ MS/MS), *H* Human (LFQ MS/MS), *D* Multiprotease digestion (LFQ MS/MS), *T *TMT analysis (MS3), *N *primary neurons (LFQ MS/MS); *F *Fly (LFQ MS/MS)

We hypothesized that by identifying proteins tightly associated with amyloid fibrils, we would be able to strengthen our understanding of how these pernicious structures are formed, cause neurotoxicity, and may be targeted for therapeutic benefit. The consequence of these proteins associating with Aβ peptides and structural assemblies causes a loss-of-function effect by reducing the pool of functional proteins. Notably, we identified IDE that degrades Aβ peptides may represent one such example [94]. On the other hand, some proteins may exhibit a gain-of-function effect when their early interaction with Aβ peptides may alter amyloid plaque formation. For example, the protein quality control machinery is likely to have a significant effect on the initiation, maturation, or stabilization of nascent amyloid seeds. However, we acknowledge that some amyloid-associated proteins likely accumulate over time in the large amyloid deposits/plaques and may not have active participation in amyloid formation, elongation, or maintenance. Such interactions may be attributed to the high hydrophobicity generated in the amyloid nanoenvironment because of the presence of fibrils/plaques in the vicinity.

In summary, ultrasonication proved to be a robust strategy to physically remove proteins weakly associated with amyloid fibrils and allowed us to comprehensively study the amyloid fibril proteome. We discovered Aβ38 in significant abundance in Aβ42-laden fibril cores, while the highly studied Aβ40 was mostly absent. While most previous reports have found that amyloid forms in the extracellular space, intracellular formation has also been found to play a key role [82, 83]. Our analysis establishes interaction between Aβ42 peptides and other proteins that are tentatively considered intracellular. Multiple experiments identified MTs in amyloid fibrils. MT3 protein was also found to be effective in triggering deposition of Aβ42 aggregates. We postulate a similar interaction pattern of other proteins identified in this analysis with Aβ42. Some of these interactions may contribute towards stability and longevity of the fibrils. Finally, we confirmed the in vivo adequacy of some of the identified proteins towards targeting Aβ42-associated toxicity in a relevant AD fly model. The genetic association was established by RNAi lines that showed a more aggressive phenotype when co-expressed with Aβ42 in the *Drosophila* eye. Remarkably, a handful of knockdown lines presented a rescue effect in the form of reduced toxicity. Future studies may help delineate more such proteins and identify modulators of Aβ42 aggregation and toxicity. We believe this work provides a foundation for more studies to identify close interaction partners and effective modulators of Aβ42 aggregation. Targeting these proteins may provide highly effective therapeutic tools to develop new AD treatments.

## Conclusions

Our novel biochemical amyloid purification strategy reduced the number of co-purifying non-specific proteins by up to three-fold. Biochemical assays confirmed presence of Aβ38 in fibrils isolated from brain and that Aβ38 can influence Aβ42 fibrilization in vitro. A comprehensive proteomic analysis identified 77 high confidence proteins that interact with Aβ42 during early deposition or formation of amyloid fibril cores. Most importantly, we identified 20 Aβ42-interacting proteins, which have never previously been reported in amyloid plaques. To test if the newly discovered fibril associated proteins play a functional role we followed up on the metal-binding protein Mt3. Interestingly, this protein, apart from showing high abundance in amyloid fibrils, modulated Aβ42 fibrilization in vitro in a metal-independent manner. Notably, knockdown of the Bcap31 fly homologue aggravated while overexpression rescued Aβ42-induced toxicity in *Drosophila* eye neurons. Similarly, overexpression of IDE (a protease) and knockdown of Serpinb5 (a protease inhibitor) also rescue toxicity in the *Drosophila* model. Overall, the results from our study identified several novel Aβ42-associated proteins that modify amyloid formation and influence neurotoxicity.

### Supplementary Information

**Additional file 1: Figure S1.** Confirmation of amyloid fibril purification. **Figure S2.** Aβ38 peptides are present in high abundance in human and mouse fibrils. **Figure S3.** Effect of Aβ38, Aβ40, and Aβ42 peptides on Aβ38 and Aβ40 amyloid fibril formation in vitro. **Figure S4.** Comprehensive MS analysis of purified mouse and human fibrils. **Figure S5.** In vitro and in vivo validation of proteomics data. **Figure S6.** Metallothionein-3, a metal-binding protein affects amyloid aggregation. **Figure S7.** Fly orthologues of MS-identified candidate proteins modulate Aβ42-induced neurotoxicity in vivo.

**Additional file 2.** Supplementary Materials and Methods.

**Additional file 3:** **Table S1.** Summary of the human subject brains. Overall, 1 control human brain tissues were used; age, gender, race and other relevant information is provided in sheet 1. For AD human samples, we obtained 13 and 23 human brain tissues with amyloid scores 2 and 3, respectively. All the relevant information are provided for each human sample, including their gender, age, race, postmortem time (PMT), clinical Braak stage, CERAD scores, etc.

**Additional file 4:** **Table S2.** List of proteins identified in label-free MS analysis of amyloid fibrils isolated from mouse cortices. Proteins identified with a higher abundance in purified fibrils (N = 8) obtained from App^NL-F/NL-F,^ and App^NL-G-F/NL-G-F^, and 5xFAD mouse brains, 6 months age, compared to respective cortex homogenate as input (n = 3 - 4). Average NSAF values for purified fibrils and cortex homogenates for individual proteins were used. Each sheet represents individual mouse genotype, and each row has individual p values using Student’s t test and adjusted *p* values using Benjamini-Hochberg (BH) correction. Number of proteins with significantly higher levels in App^NL-F/NL-F^, and App^NL-G-F/NL-G-F,^ and 5xFAD brains are 59, 32, and 29, respectively. Experiment = specific data set, Uniprot accession = Uniprot identifier for each protein, ratio = log2 average NSAF values (purified fibrils/homogenate), t test *p* value = t test *p* value, Rank = rank ordered proteins based on *p* value (if *p* values are identical, higher ratio was listed first). Additional remarks indicate if results (increased abundance in purified fibrils) are statistically significant or not.

**Additional file 5:** **Table S3.** List of proteins identified in label free MS analysis of amyloid fibrils isolated from human AD cortices. Proteins with significantly higher abundance in purified fibrils obtained from human brain cortices, with amyloid (A) score 2 and 3 (*N*= 13 and 23 patients respectively), compared to respective cortex homogenate as input (*N* = 4). NSAF values for purified fibrils and cortex homogenates for individual proteins were used. Separate sheets are provided for A2 and A3 brain amyloid fibrils data sets, and each row has individual *p* values using Student’s t test, followed by adjusted *p* values with BH correction. Number of significantly high abundance proteins in A2 and A3 fibril cores are 252 and 330, respectively. Experiment = specific data set, Uniprot accession = Uniprot identifier for each protein, ratio= log2 average NSAF values (purified fibrils/homogenate), t test *p* value = t test *p* value, Rank = rank ordered proteins based on *p* value (if *p* values are identical, higher ratio was listed first), description = protein description. Additional remarks indicate if results (increased abundance in purified fibrils) are statistically significant or not.

**Additional file 6:** **Table S4.** List of proteins identified in label-free MS analysis of amyloid fibrils isolated from mouse and human cortices following multiprotease digestion. Proteins identified in purified fibrils following their additional multiprotease digestion (*N* = 8 - 10). Each sheet represents an individual mouse line or human patient data sets, and each row has individual *p* values using Student’s t test, and adjusted *p* values with BH correction. Experiment = specific data set, Uniprot accession= Uniprot identifier for each protein, ratio= log2 average NSAF values (purified fibrils/homogenate), t test *p* value = t test *p* value, Rank= rank ordered proteins based on *p* value (if *p* values are identical, higher ratio was listed first), Adjusted *p* value is calculated using BH correction, description= protein description.

**Additional file 7:** **Table S5.** List of proteins identified in multiplex TMT analysis of amyloid fibrils isolated from mouse of different age groups. Proteins identified in 16-plex TMT analysis containing eight biological conditions, each in two biological replicates. Average normalized TMT intensity values have been used for making comparisons between individual conditions. No intensity cutoff is applied. Each sheet shows comparisons between individual biological groups and proteins only in higher abundance in every comparison shown in the table. Experiment = specific data set, Uniprot accession= Uniprot identifier for each protein, ratio= log2 average TMT intensity (group 1/group 2), protein= protein name, description= protein description.

**Additional file 8: Table S6.** List of proteins identified in label-free MS analysis of amyloid fibrils isolated from rat hippocampal and cortical neurons incubation recombinant Aβ42 seeds. Proteins identified in fibrils purified from rat cortical and hippocampal neurons (*n *= four). Proteins identified in least two independent fibril preparations from each culture type (> 4 total, at least 2 cortex + 2 hippocampal) were considered and the table shows only those proteins that were also identified in the TMT analysis of purified amyloid from mouse brains (shown in Table S5). Experiment = specific data set, Uniprot accession = Uniprot identifier for each protein, occurrence score = number of occurrences in independent fibril preparations, description = protein description.

**Additional file 9: Table S7.** List of proteins identified in label-free MS analysis of amyloid fibrils isolated from Aβ42 fly heads. Proteins identified with significantly higher levels in purified fibrils obtained from Aβ42 flies, compared to control Lac-Z flies (*N* = four independent biological replicates). In sheet 1, NSAF values for purified fibrils and cortex homogenates for individual proteins were used, each row has individual *p* values using Student’s t test, followed by adjusted *p* values with BH correction. Corresponding human and mouse orthologs have been identified for each fly gene (https://www.flyrnai.org/diopt). Sheet 2 shows proteins considered for obtaining RNAi lines following identification of Fly orthologs based on scoring. Sheet 3 and 4 indicates individual severity score and all the analysis performed, Sheet 5 and 6 indicates orthologous human and mouse genes, respectively. Experiment = specific data set, Uniprot accession= Uniprot identifier for each protein, ratio= log2 average NSAF values of purified fibrils (Aβ42 /control flies), protein = protein name, t test *p* value = t test *p* value, Rank = rank ordered proteins based on *p* value (if *p* values are identical, higher ratio was listed first), Adjusted *p* value is calculated using BH correction, description= protein description, putative human ortholog = human gene orthologous to the identified fly genes, putative mouse ortholog = mouse gene orthologous to the identified fly genes obtained using Dipot online tool, only high and moderate scoring genes were considered in the analysis). Additional remarks indicate if results (increased abundance in purified fibrils) are statistically significant or not.

## Data Availability

All data are available in the main text or the supplementary information files. Experimental procedures, methods of data collection and analysis are provided in Additional file 2. The analyzed MS datasets for individual MS experiments are provided in supplementary Tables S1, S2, S3, S4, S5 and S6. All raw mass spectrometry data can be accessed on MassIVE and Proteome Exchange under MSV000092311.

## Abbreviations

Aβ: Amyloid beta
AD: Alzheimer’s disease
ARF5: ADP ribosylation factor 5
BCAP31: B-cell receptor-associated protein 31
CERAD: Consortium to establish a registry for Alzheimer's disease
CR: Congo red
DDX3Y: DEAD box protein 3, Y-chromosomal
ELISA: Enzyme-linked immunoassay
GSM: Gamma-secretase modulators
HMW: High molecular weight
HSP70: Heat shock protein 70
IDE: Insulin degradation enzyme
IDH3B: Isocitrate dehydrogenase
LC-MS: Liquid chromatography- mass spectrometry
LFQ: Label-free quantification
LMW: Low molecular weight
MT: Metallothionein
PPIA: Peptidyl-prolyl cis–trans isomerase A
SRSF4: Serine and arginine rich splicing factor 4
STXBP1: Syntaxin-binding protein 1
ThT: Thioflavin T
TMT: Tandem mass tag
UCHL1: Ubiquitin C-terminal hydrolase L1
