# Supplementary material for: Amyloid fibril proteomics of AD brains reveals modifiers of aggregation and toxicity
Source: Mol Neurodegener. 2023 Sep 14;18:61. doi: 10.1186/s13024-023-00654-z (PMC10503190; doi:10.1186/s13024-023-00654-z)
Supplement: Supplementary file 1 — Additional file 1: Figure S1. Confirmation of amyloid fibril purification. Figure S2. Aβ38 peptides are present in high abundance in human and mouse fibrils. Figure S3. Effect of Aβ38, Aβ40, and Aβ42 peptides on Aβ38 and Aβ40 amyloid fibril formation in vitro. Figure S4. Comprehensive MS analysis of purified mouse and human fibrils. Figure S5. In vitro and in vivo validation of proteomics data. Figure S6. Metallothionein-3, a metal-binding protein affects amyloid aggregation. Figure S7. Fly orthologues of MS-identified candidate proteins modulate Aβ42-induced neurotoxicity in vivo. [file 13024_2023_654_MOESM1_ESM.docx]

**SUPPLEMENTARY INFORMATION FILE 1**

**Supplementary Figures and Tables**

**
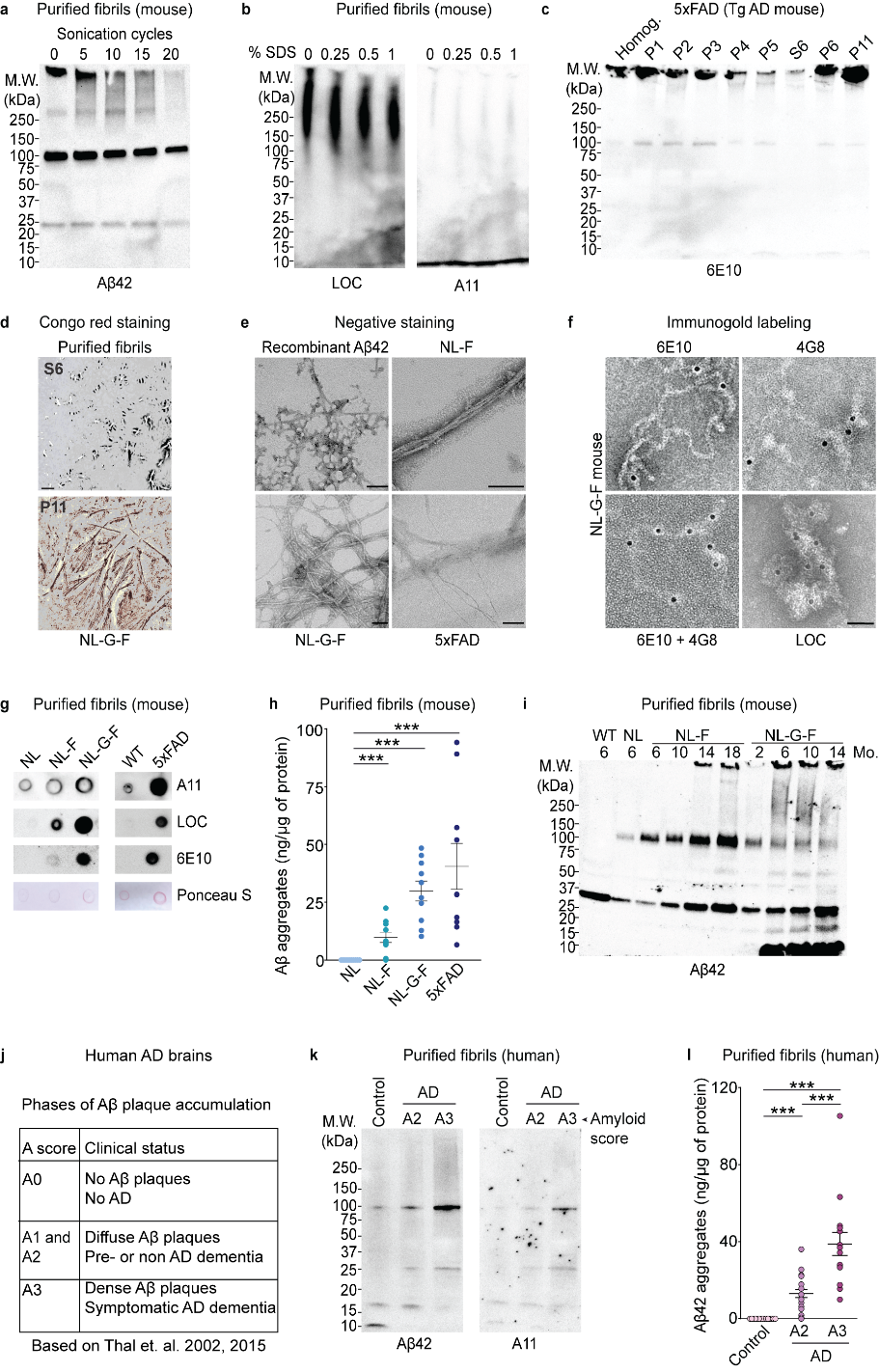
**

**Figure S1. Confirmation of amyloid fibril purification.**

(a) Representative Western blot (WB) confirming effects of ultrasonication on high molecular weight (HMW) fibrils isolated from 5XFAD brains.

(b) Western blot analysis of SDS-resistant fibril species isolated from 5XFAD brains.

(b) Representative WB analysis of SDS treated (indicated %s, 1 hour incubation) purified fibrils isolated from 5XFAD brains.

(c) Representative Aβ_1-16_ (6E10) WB across indicated fractions of transgenic 5xFAD cortical extracts collected during fibril purification. The membrane shown in Figure 1b was stripped and re-probed with 6E10 antibody.

(d) Amyloid specific Congo red (CR) staining of fibril cores. Bright field images of the P11 fraction from NL-G-F mouse cortical extracts stained with amyloid-specific CR. SDS soluble (S6) fraction is used as a control.

(e) Representative negative staining electron micrographs for *App^NL-F/NL-F^*, *App^NL-G-F/NL-G-F^* and 5xFAD fibrils and recombinant Aβ42 peptide fibrils as positive control.

(f) Panels of negative staining EM images showing immunogold labeling of NL-G-F fibrils using Aβ_1-16_ (6E10), Aβ_17-24_ (4G8), and anti-fibril (LOC) antibodies.

(g) Dot blot analysis of three *App KI* (*App^NL/NL^*, *App^NL-F/NL-F^*, and *App^NL-G-F/NL-G-F^*); WT and transgenic 5xFAD brain amyloid fibrils using LOC, A11 and 6E10 antibodies.

(h) Sandwich ELISA-based quantification of Aβ aggregates in purified amyloid material extracted from indicated mouse brains.

(i) Representative WB indicating abundance of Aβ42 monomers and higher order assemblies in purified amyloid extracted from all three *App KI* (*App^NL/NL^*, *App^NL-F/NL-F^*, and *App^NL-G-F/NL-G-F^*) brains and WT control at the indicated age.

(j) A brief summary of clinical stages of amyloid pathology and amyloid scoring of human brains. The detailed scoring guidelines and clinical criteria for A scores are available in Thal et al. 2002, 2015 ^1,2^.

(k) WB analysis of purified amyloid assemblies prepared from human brains using to probe Aβ42 and A11 antibodies.

(l) Quantification of total Aβ aggregates in human amyloid extracts using sandwich ELISA. Fifteen amyloid samples from each indicated groups were analyzed.

Data in h and l represents mean ± SEM; *, p-value < 0.05; **, p-value< 0.01; ***, p-value < 0.001; analyzed with unpaired Student’s t-test or one-way ANOVA with post-hoc Sidek test. 6E10 antibody detects Aβ_1-16;_ 4G8 antibody detects Aβ_17-24;_ Tg = transgenic. P = pellet, S = supernatant. NL *= App^NL/NL^*, NL-F *= App^NL-F/NL-F^*, NL-G-F = *App^NL-G-F/NL-G-F^*; N = 3 (a, b, d, f, and k), 5 (c, e, and g), 10 mice (h), and N = 15 humans (l). All mice were 6 months of age. Scale bar = 100 µm (d), 10 µm (e), 500 nm (f), 50 nm (g). Scale bar = 10 µm (c), 100 nm (d), and 50 nm (e).

**
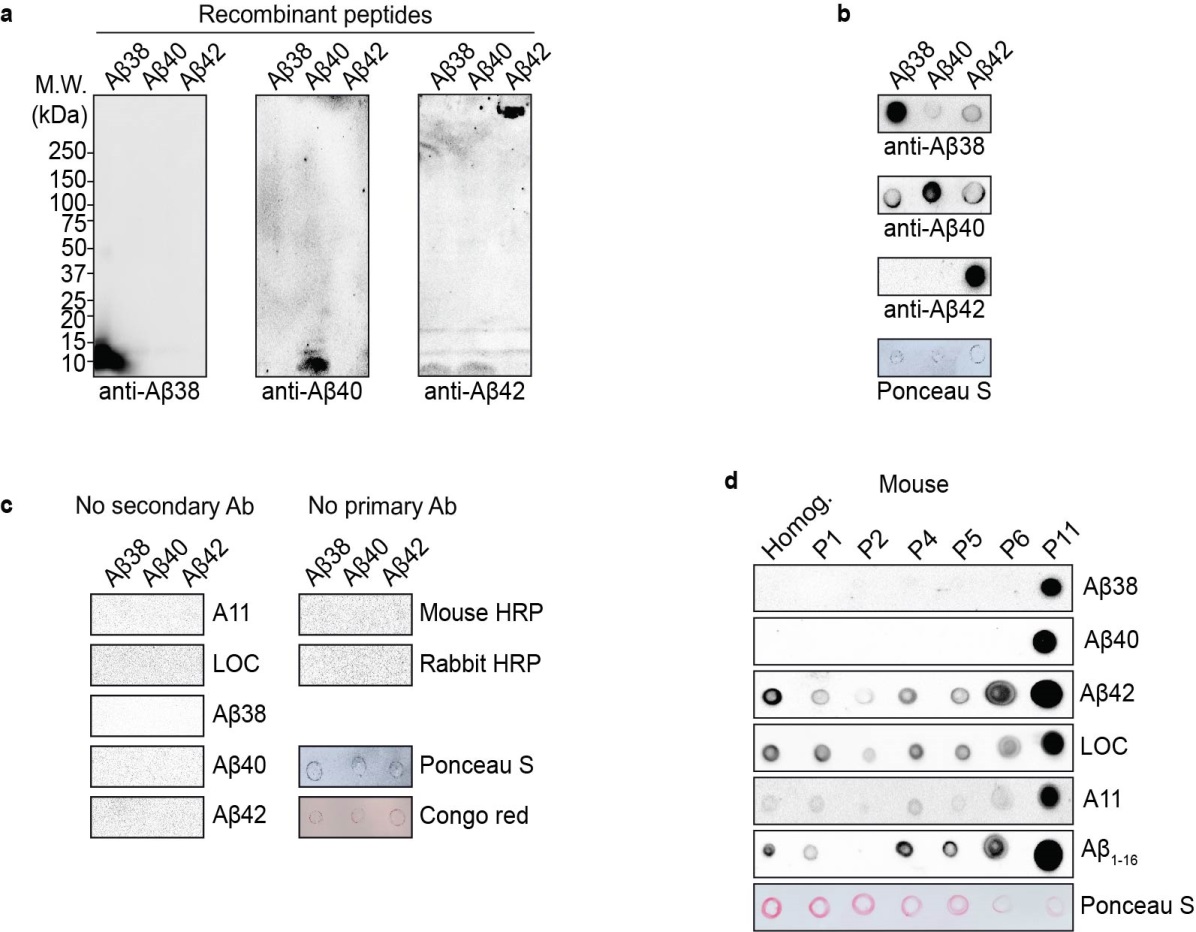
**

**Figure S2. Aβ38 peptides are present in high abundance in human and mouse fibrils**

(a-b) WB analysis and dot blots of recombinant Aβ38, Aβ40 and Aβ42 peptides using respective antibodies. Ponceau S stained membranes (b) were used for visualization of loading protein amount.

(c) Dot blot analysis using only primary antibody or only secondary antibodies. Ponceau S and Congo red stained membranes were used to visualize total protein amount.

(d) Dot blot analysis of indicated fractions collected during amyloid fibril purification from *App^NL-G-F/NL-G-F^* mouse cortex using monomer-specific anti-Aβ38, Aβ40 and Aβ42 and conformation-specific antibodies. Ponceau S-stained membranes were used for visualization of loading protein amount. N = 5 (a, b, and d), N = 3 (c).

**
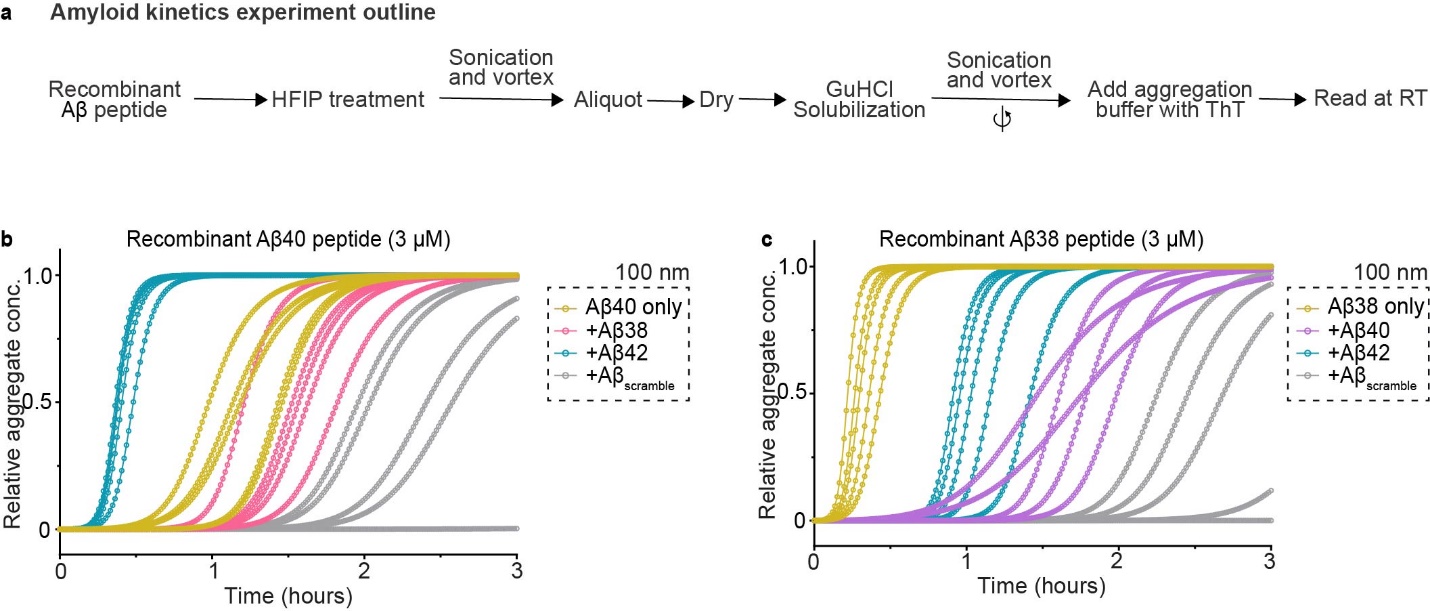
**

**Figure S3. Effect of Aβ38, Aβ40, and Aβ42 peptides on Aβ38 and Aβ40 amyloid fibril formation in vitro.**

(a) Aβ peptide solubilization, monomerization, and assessment of fibril formation based on Thioflavin T (ThT)-based amyloid kinetic analysis. RT = room temperature.

(b-c) ThT-based amyloid kinetics of two-peptide system consisting of Aβ38 or Aβ40 (3 μM) in absence or presence of Aβ38, Aβ40, Aβ42, and Aβ42_scramble_ peptides (100 nM). The relative amyloid concentrations were calculated using secondary nucleation model in AmyloFit online tool (https://amylofit.com/amylofitmain). ThT fluorescence intensities were measured every four minutes. N = 5 (b, and c).

**
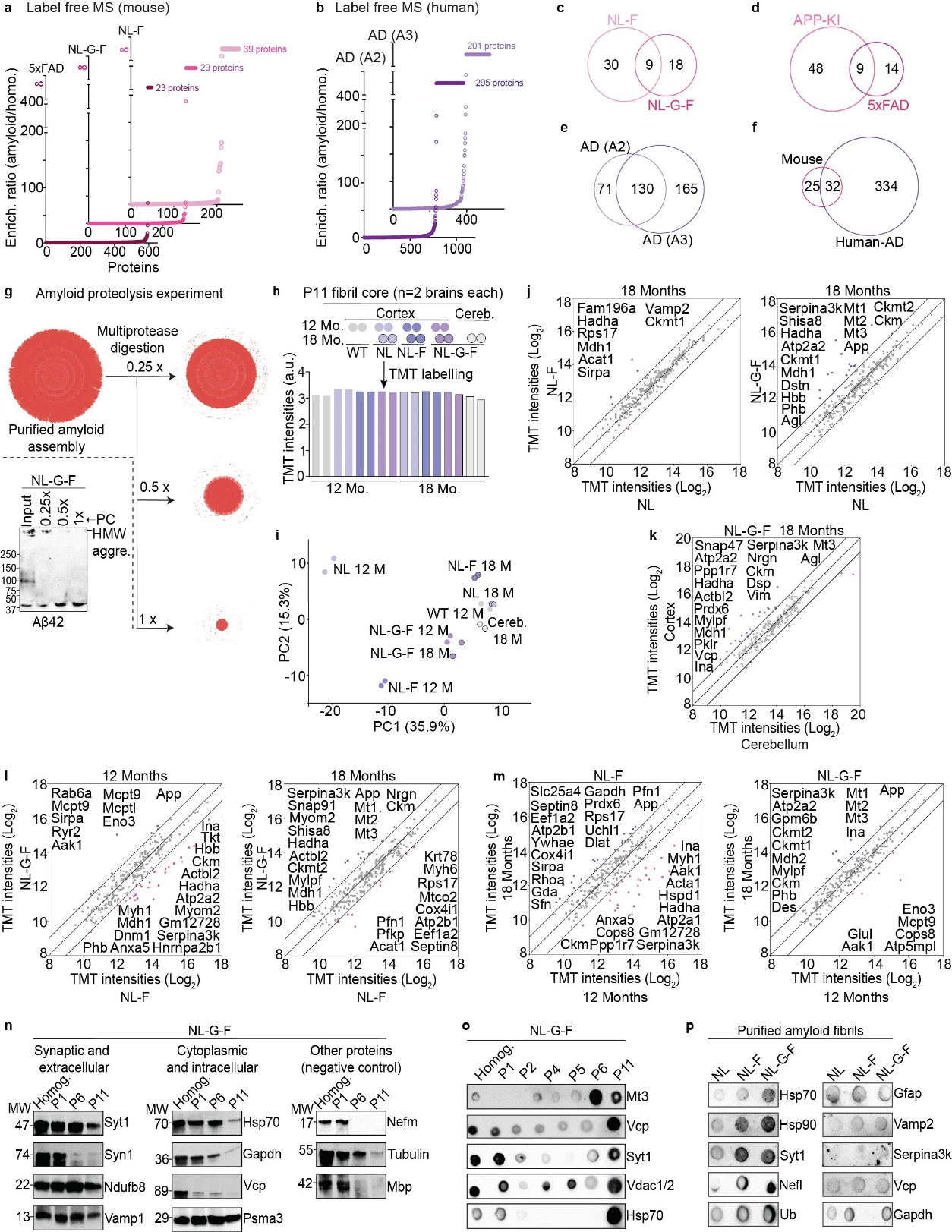
**

**Figure S4. Comprehensive MS analysis of purified mouse and human fibrils**

(a-b) Rank order plots of proteins for ratios between average NSAF values in purified fibrils compared to cortex homogenate samples in a label free MS analysis for indicated mouse and human fibril extracts. Fibrils obtained from eight mouse cortices for each group; 16 control,13 A2, and 23 A3 human AD brains analyzed. Label free MS data from four brain homogenates for each groups were obtained for comparison and analysis.

(c-d) Venn diagram comparing proteins exclusively identified in purified amyloids prepared from indicated mouse strains.

(e) Venn diagram comparing proteins exclusively identified in purified amyloid fibrils prepared from postmortem human AD (A2 and A3) brains.

(f) Venn diagram indicating number of proteins identified purified fibrils extracted from both human AD and mouse (*App^NL-F/NL-F^*, *App^NL-G-F/NL-G-F^*, 5xFAD) brains.

(g) Outline of multiprotease digestion experiment; representative blot in the inset showing concentration-dependent degradation of HMW amyloid assemblies following treatment with cocktail of multiple proteolytic enzymes: Arg-C, Asp-N, Lys-C, Glu-C, thermolysin, chymotrypsin, and trypsin.

(h) Experimental setup and relative TMT intensities across the 16-plex TMT channels. Two biological replicates were pooled for each TMT channel.

(i) PCA analysis of different biological samples using normalized TMT intensities of all identified proteins.

(j-m) Scatter plots comparing average TMT intensities of proteins measured in fibril cores extracted from 12- or 18-months old *App KI* mouse cortices. A comparison of proteins identified in cortical and cerebellar fibrils is plotted in k.

(n-o) Representative WBs and dot blot analysis of indicated fractions collected during amyloid fibril purification probing top protein candidates identified in MS analyses. Few proteins (either not detected or non-specifically bound to fibrils) were immunoblotted as negative controls.

(p) Representative dot blots of relative levels of proteins in P11 fractions obtained from three *App KI* brains. P = pellet, and S = supernatant. *NL = App^NL/NL^*, *NL-F = App^NL-F/NL-F^*, *NL-G-F* = *App^NL-G-F/NL-G-F^*. N = 8 mice (a, c, d); N = 15 control, 13 AD A2, 23 AD A3 humans (b, e, and f).

**
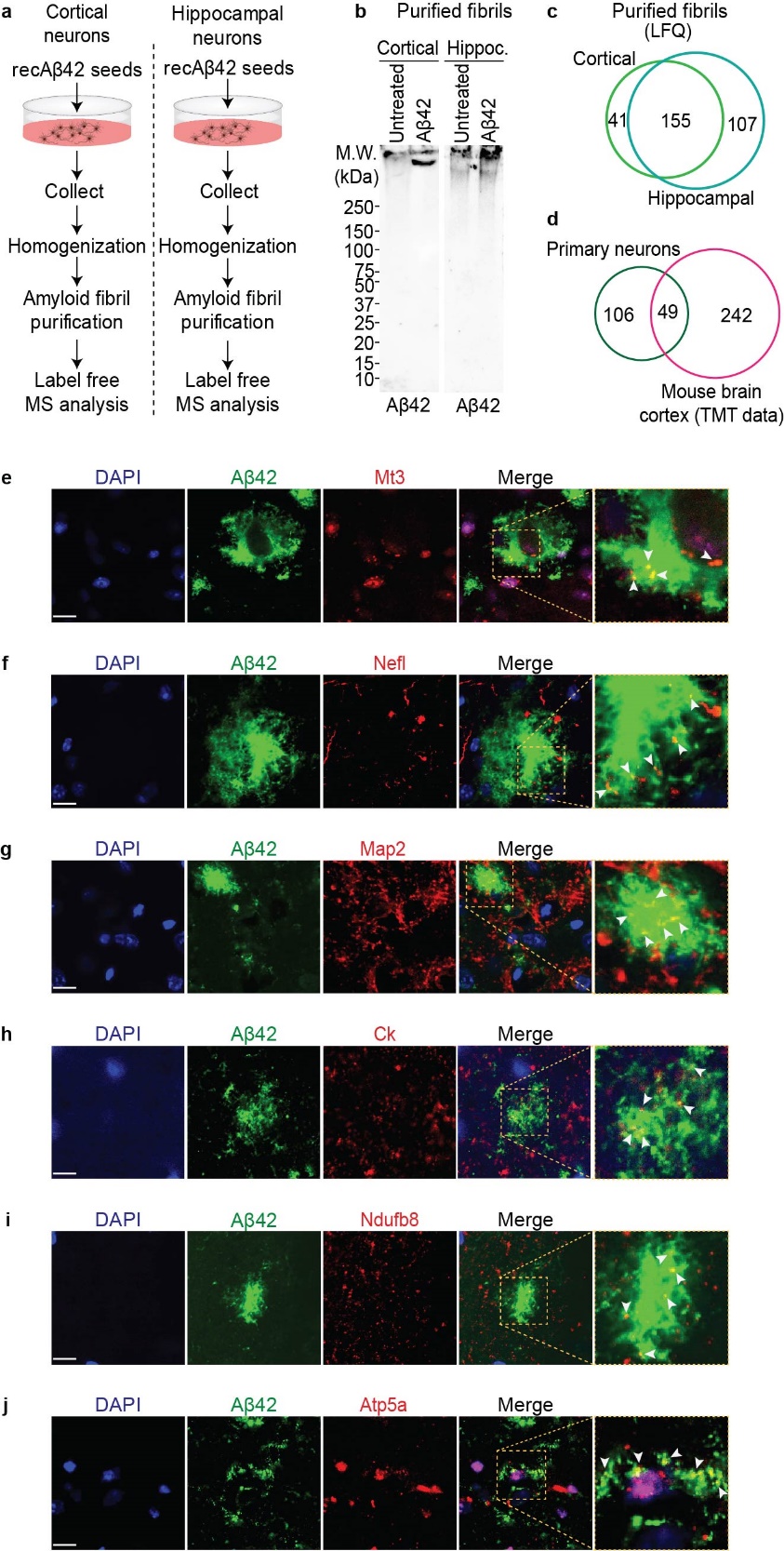
**

**Figure S5. *In vitro* and *in vivo* validation of proteomic data**

1. Experimental outline of amyloid purification and supplementary MS analysis of fibrils collected from primary rat neurons seeded with recombinant Aβ42 peptides.

(b) Representative WB confirming formation of Aβ42-containing HMW aggregates in seeded neurons in comparison to unseeded neurons.

(c) Venn diagram comparing proteins identified in fibrils isolated from Aβ42 seeded hippocampal and cortical neurons

(d) Venn diagram comparing proteins identified in neurons and those identified in fibrils purified from mouse cortex (TMT experiment dataset).

(e-j) Representative confocal images of IHC staining of three months old NL-G-F mouse brain cortex for most common proteins identified in multiple MS analysis confirming colocalization with large amyloid plaques stained with anti-Aβ42 antibody.

N= 4 replicates (c); Scale bar: 10 μm (e-j).

**
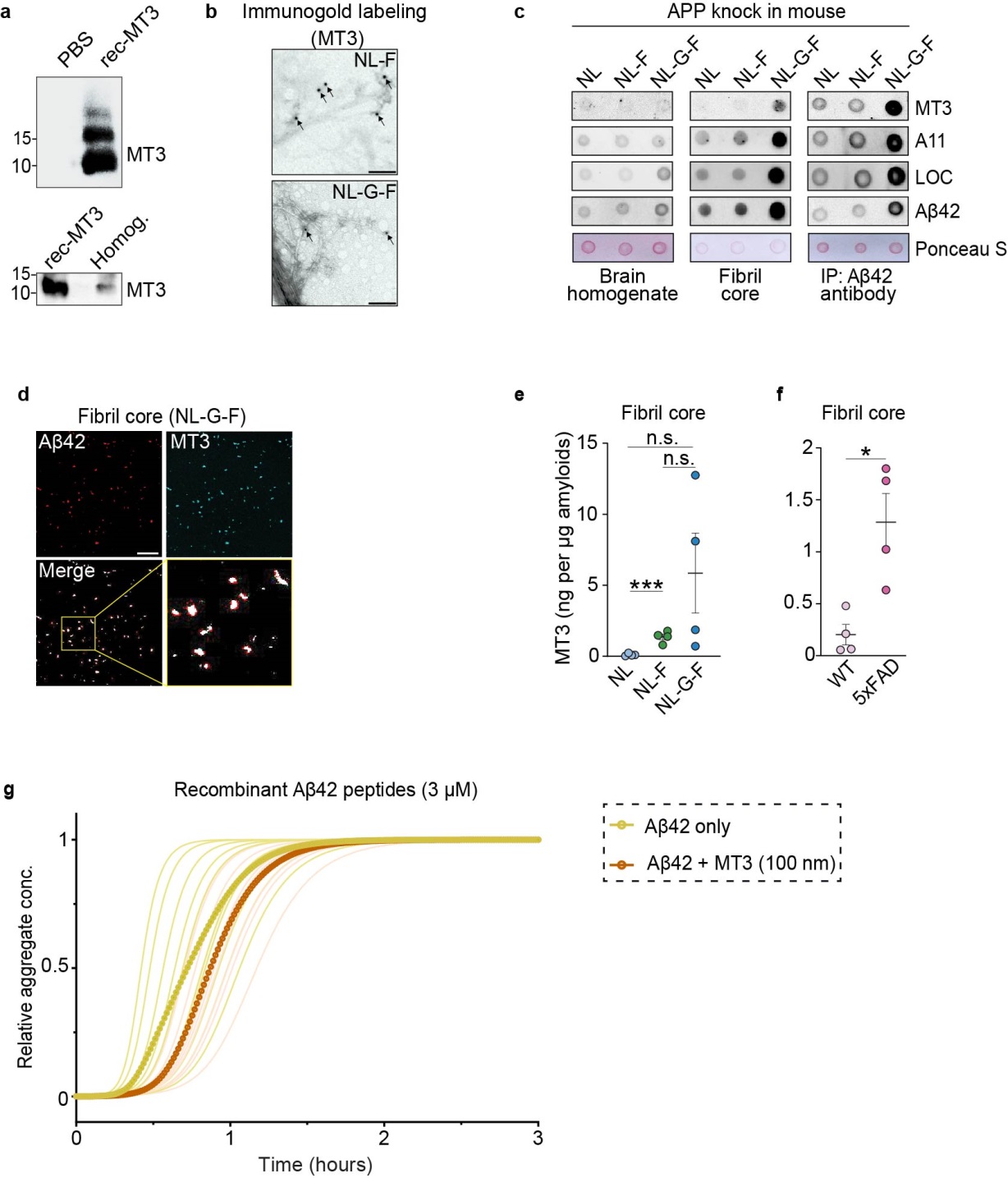
**

**Figure S6. Metallothionein-3, a metal-binding protein affects amyloid aggregation**

(a) WB-based assessment of Mt3 antibodies.

(b) Representative immunogold labelling EM image panels confirming presence of Mt3 in purified amyloid fibrils isolated from *NL-F* and *NL-G-F* brains.

(c) Dot blot analysis of Mt3 protein in *App KI* brain homogenates, purified fibrils, and affinity purified Aβ42. Ponceau S-stained membranes were used for visualization of loading protein amount.

(d) Monoclonal antibody based analysis of Mt3 and Aβ42 in purified fibrils spotted on glass slides.

(e-f) Sandwich ELISA for Mt3 protein in purified fibrils isolated from indicated mouse strains.

(g) ThT based *in vitro* aggregation kinetics experiment shows varying effects of 100 nM of Mt3 protein on the relative aggregation of 3 μM recombinant Aβ42 peptides. N = 10.

*NL = App^NL/NL^*, *NL-F = App^NL-F/NL-F^*, *NL-G-F* = *App^NL-G-F/NL-G-F^*. Data in e-f represents mean ± SEM; *, p-value <.05; **, p-value< .01; ***, p-value < .001 analyzed with unpaired Student’s t-test or one-way ANOVA with post hoc Sidak test. N= 3 (c, d), 4 mice (e, f), 10 (g); Scale bar = 100 nm (b), 10 µm (d).

**
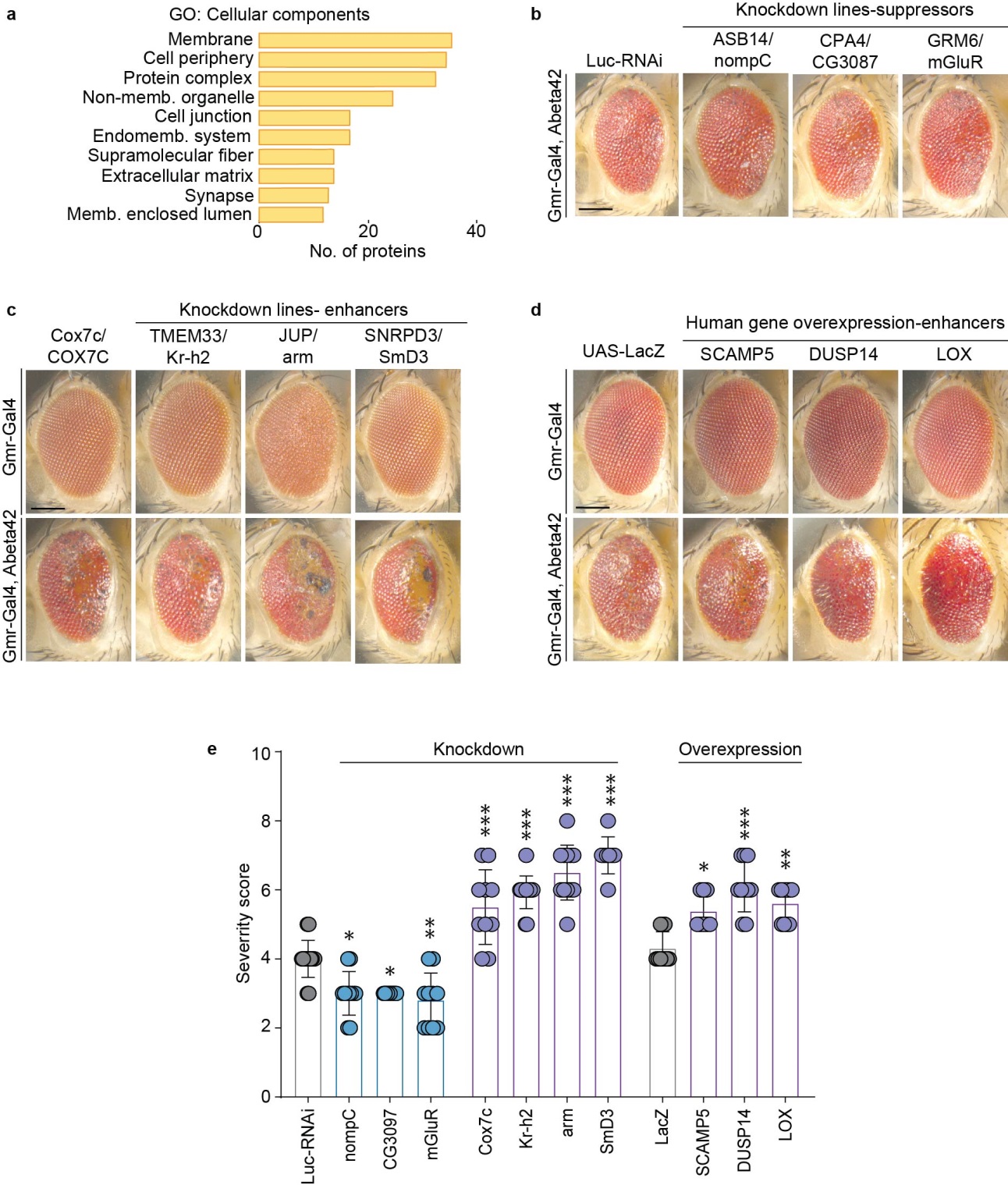
**

**Figure S7. Fly orthologues of MS-identified candidate proteins modulate Aβ42-induced neurotoxicity in vivo**

(a) GO enrichment analysis: cellular components for genes significantly enriched in Aβ42 flies compared to control flies showing abundance of membranous, extracellular, and synaptic proteins in amyloid fibrils.

(b) Representative eye images showing suppression of Aβ42-induced eye phenotypes upon expression of the indicated RNAi knockdown lines.

(c) Representative eye images showing enhancement of Aβ42-induced eye phenotypes upon expression of the indicated RNAi lines. Note that these lines have little to no effect on eye morphology in the absence of Aβ42 (top row).

(d) Representative eye images showing that overexpression of the indicated human transgenes enhances Aβ42-mediated phenotypes in the fly eye compared to the effect of the innocuous LacZ control transgene.

These human genes do not disrupt the eye structures in the absence of Aβ42 (top row).

(e) Quantification of (b-d).

Data in e represents mean ± SD; **, p-value< .01; ***, p-value < .001; analyzed with ordinary one-way ANOVA followed by Dunnett’s multiple comparison test. N = 8-15 flies per line (e). Scale bar = 100 µm (b, c, d).

**References**

1. Thal, D.R., Rüb, U., Orantes, M. & Braak, H. Phases of A beta-deposition in the human brain and its relevance for the development of AD. *Neurology* **58**, 1791-800 (2002).

2. Thal, D.R., Walter, J., Saido, T.C. & Fändrich, M. Neuropathology and biochemistry of Aβ and its aggregates in Alzheimer’s disease. *Acta Neuropathologica* **129**, 167-182 (2015).
